# Author Correction: Multi-scale classification decodes the complexity of the human E3 ligome

**DOI:** 10.1038/s41467-026-69474-1

**Published:** 2026-02-10

**Authors:** Arghya Dutta, Alberto Cristiani, Siddhanta V. Nikte, Jonathan Eisert, Yves Matthess, Borna Markusic, Cosmin Tudose, Chiara Becht, Ronay Cetin, Varun Jayeshkumar Shah, Thorsten Mosler, Koraljka Husnjak, Ivan Dikic, Manuel Kaulich, Ramachandra M. Bhaskara

**Affiliations:** 1 Institute of Biochemistry II, Faculty of Medicine, Goethe University, Theodor-Stern-Kai 7, Frankfurt am Main, Germany; 2 Buchmann Institute for Molecular Life Sciences, Goethe University, Max-von-Laue Str. 15, Frankfurt am Main, Germany; 3 Department of Physics, SRM University–AP, Amaravati, Andhra Pradesh India; 4 IMPRS on Cellular Biophysics, Max-von-Laue Str. 3, Frankfurt am Main, Germany

**Keywords:** Data integration, Target identification, Machine learning, Ubiquitylation, Ligases

Correction to: *Nature Communications* 10.1038/s41467-025-67450-9, published online 25 December 2025

In the version of the article initially published, Ronay Cetin (Institute of Biochemistry II, Faculty of Medicine, Goethe University, Theodor-Stern-Kai 7, Frankfurt am Main, Germany) did not appear in the author list and is now included in the HTML and PDF versions of the article.

