# Supplementary material for: Multi-scale classification decodes the complexity of the human E3 ligome
Source: Nat Commun. 2025 Dec 25;16:11382. doi: 10.1038/s41467-025-67450-9 (PMC12741051; doi:10.1038/s41467-025-67450-9)
Supplement: Supplementary file 1 — Supplementary Information [file 41467_2025_67450_MOESM1_ESM.pdf]

Supplementary material

for

Multi-scale classification decodes the complexity of the human E3 ligome

Arghya Dutta[1,2,3†], Alberto Cristiani[1,2†], Siddhanta V. Nikte[1,2†], Jonathan Eisert[1,2], Yves Matthess[1], Borna Markusic[1,4], Cosmin Tudose[1], Chiara Becht[1], Varun Jayeshkumar Shah[1], Thorsten Mosler[1], Koraljka Husnjak[1], Ivan Dikic[1,2,4], Manuel Kaulich[1], Ramachandra M. Bhaskara[1,2,4*]

[1]Institute of Biochemistry II, Faculty of Medicine, Goethe University, Theodor-Stern-Kai 7, 60590 Frankfurt am Main, Germany.

[2]Buchmann Institute for Molecular Life Sciences, Goethe University, Max-von-Laue Str. 15, 60438 Frankfurt am Main, Germany.

[3] Department of Physics, SRM University-AP, Amaravati 522240, Andhra Pradesh, India.

[4]IMPRS on Cellular Biophysics, Max-von-Laue Str. 3, 60438, Frankfurt am Main, Germany.

[*]**Corresponding author:** R.M.B; **E-mail:** bhaskara@med.uni-frankfurt.de

[†]These authors contributed equally to this work.

This PDF file includes:

- Supplementary Figures (1–23)
- Supplementary Tables (1–7)
- Supplementary Notes (1–17)
- Supplementary References

Supplementary Figures

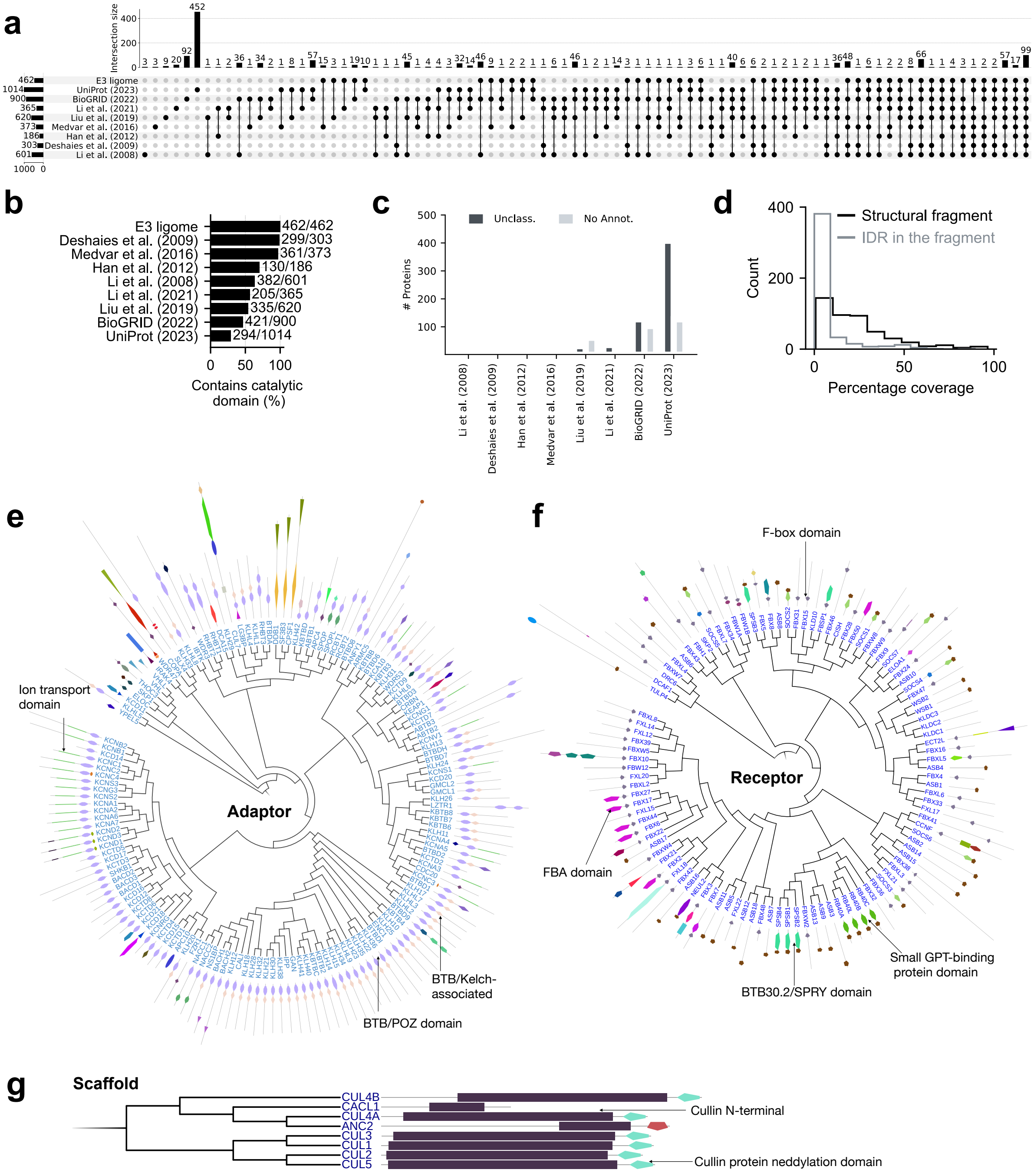

Figure S1: **Comparison of E3 ligase datasets. (a)** Complete UpSet plot showing overlap of 8 different E3 ligase datasets along with the curated E3 ligome. **(b)** Fraction of proteins (%) containing catalytic domains across various E3 datasets. **(c)** Distribution of unclassified and unannotated proteins across source datasets. **(d)** Sequence coverage (%) of structural fragments (pLDDT$\geq$ 50; black) and intrinsically disordered regions (pLDDT$<$ 50; gray) within the E3 ligome. Unrooted dendrograms showing hierarchical clusters of non-catalytic E3 components, **(e)** adaptor (n = 151), **(f)** receptor (n = 106) and **(g)** scaffold proteins (n = 8). Individual leaves show domain architectures (scaled by protein size) with annotated domains.

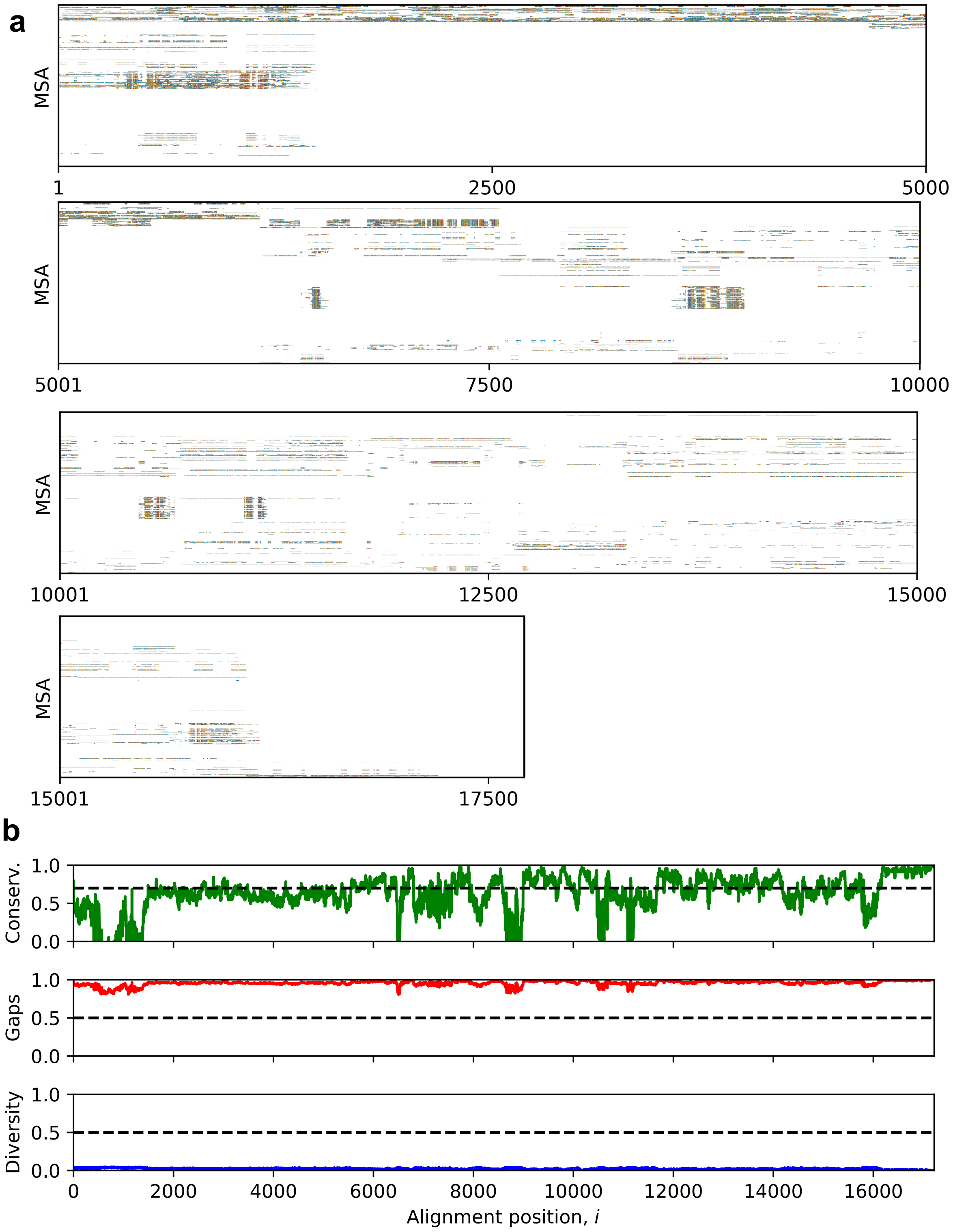

Figure S2: **Multiple sequence alignment of the E3 ligome.** **(a)** Full-length MSA of all catalytic components of the E3 ligome (n = 462 E3s) using T-Coffee. Indels or gaps (white-blank spaces) are prevalent and interspersed with sparsely aligned regions (colored). **(b)** The residue conservation (green, top), indel or gap frequency (red, middle), and amino-acid diversity (blue, bottom) at each alignment position i are far from the threshold values (dashed black lines), indicating poor, unreliable alignment features.

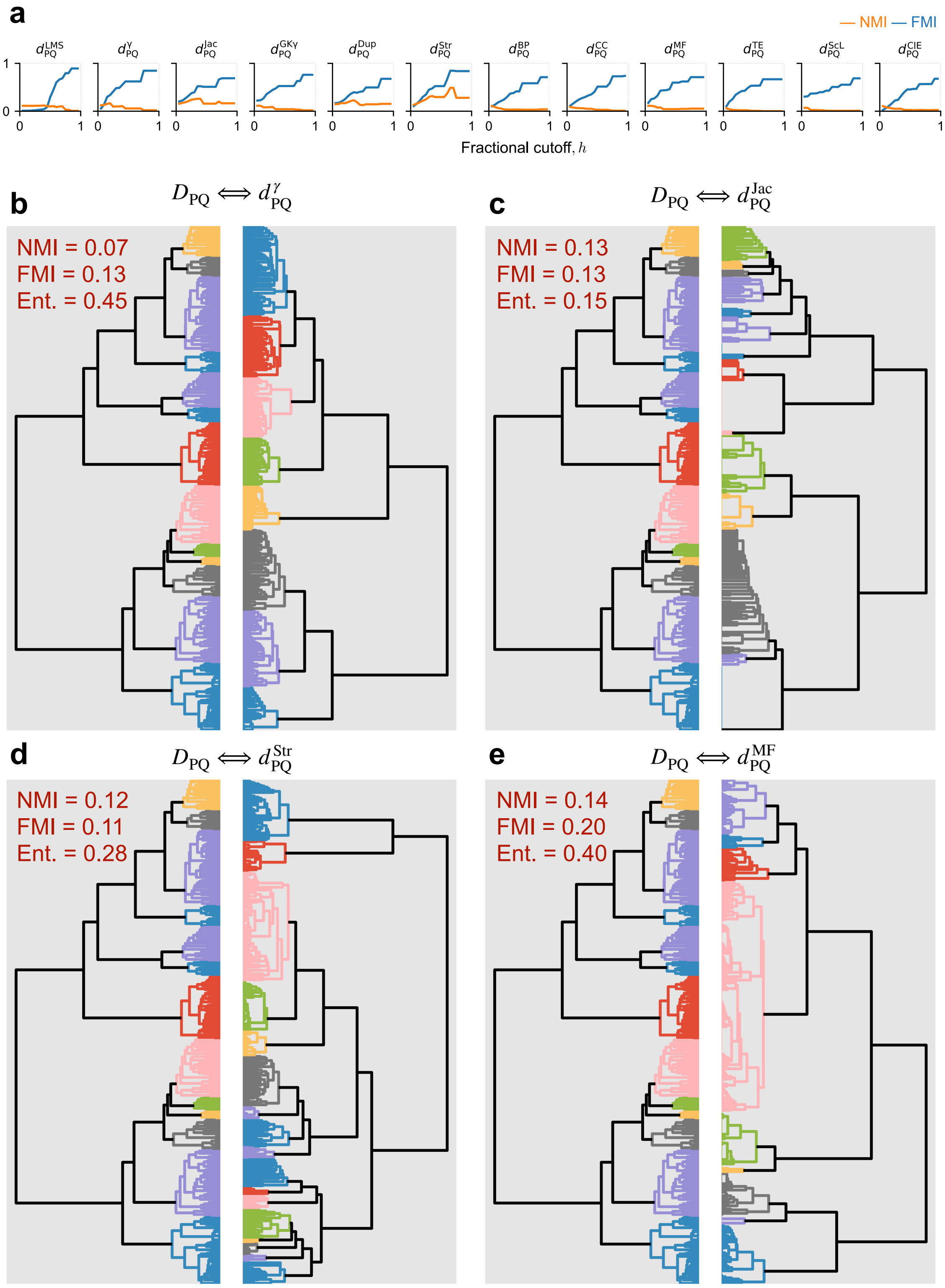

Figure S3: **Alternate cluster-comparison metrics.** **(a)** Normalized mutual information (NMI, orange) and Fowlkes-Mallows index (FMI, blue), which compare clusters from various distances and the ground truth, are sensitive to cluster count and fail to penalize unified clusters ($h \approx 1$) or singleton clusters ($h \approx 0$), respectively. Pair-wise comparison of hierarchical trees from D_{PQ} (left) and single distance measures (right) using **(b)** d_{PQ}^{γ}, **(c)** d_{PQ}^{Jac}, **(d)** d_{PQ}^{Str}, and **(e)** d_{PQ}^{MF}. Distinct clusters at $h = 0.25$ are colored. NMI, FMI, and entanglement (Ent.) scores (top-left) provide quantification for the comparisons.

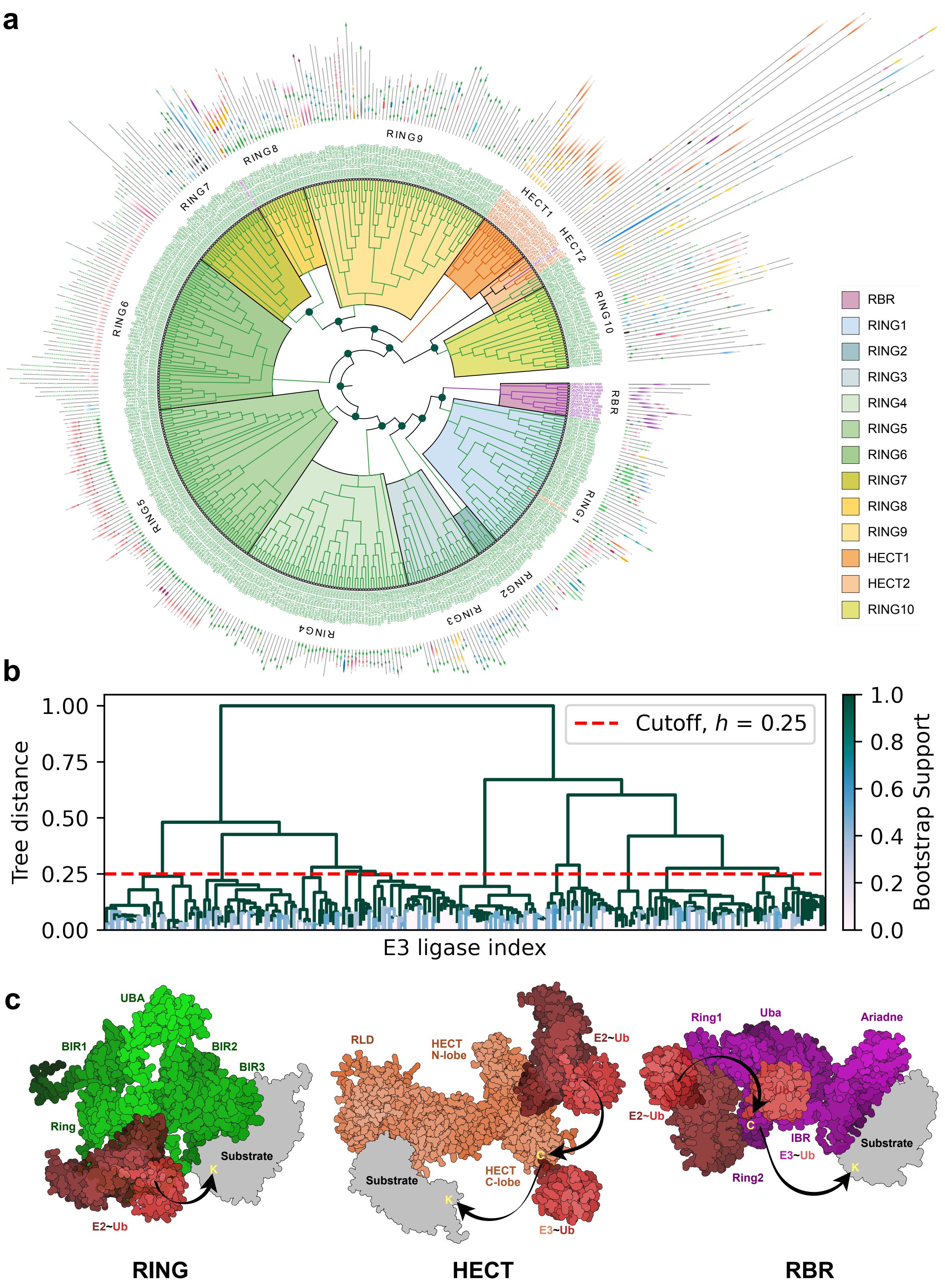

Figure S4: **E3 ligome classes and families. (a)** Circular representation of the emergent hierarchical tree displaying individual classes and families (colored) along with domain architecture information mapped onto individual leaves. **(b)** Corresponding dendrogram (scaled) showing bootstrap support for all the nodes after resampling (n = 500) the optimized distance matrix, D_{PQ}. **(c)** Schematics of Ub-transfer mechanisms for RING, HECT, and RBR classes; Atypical mechanisms not shown.

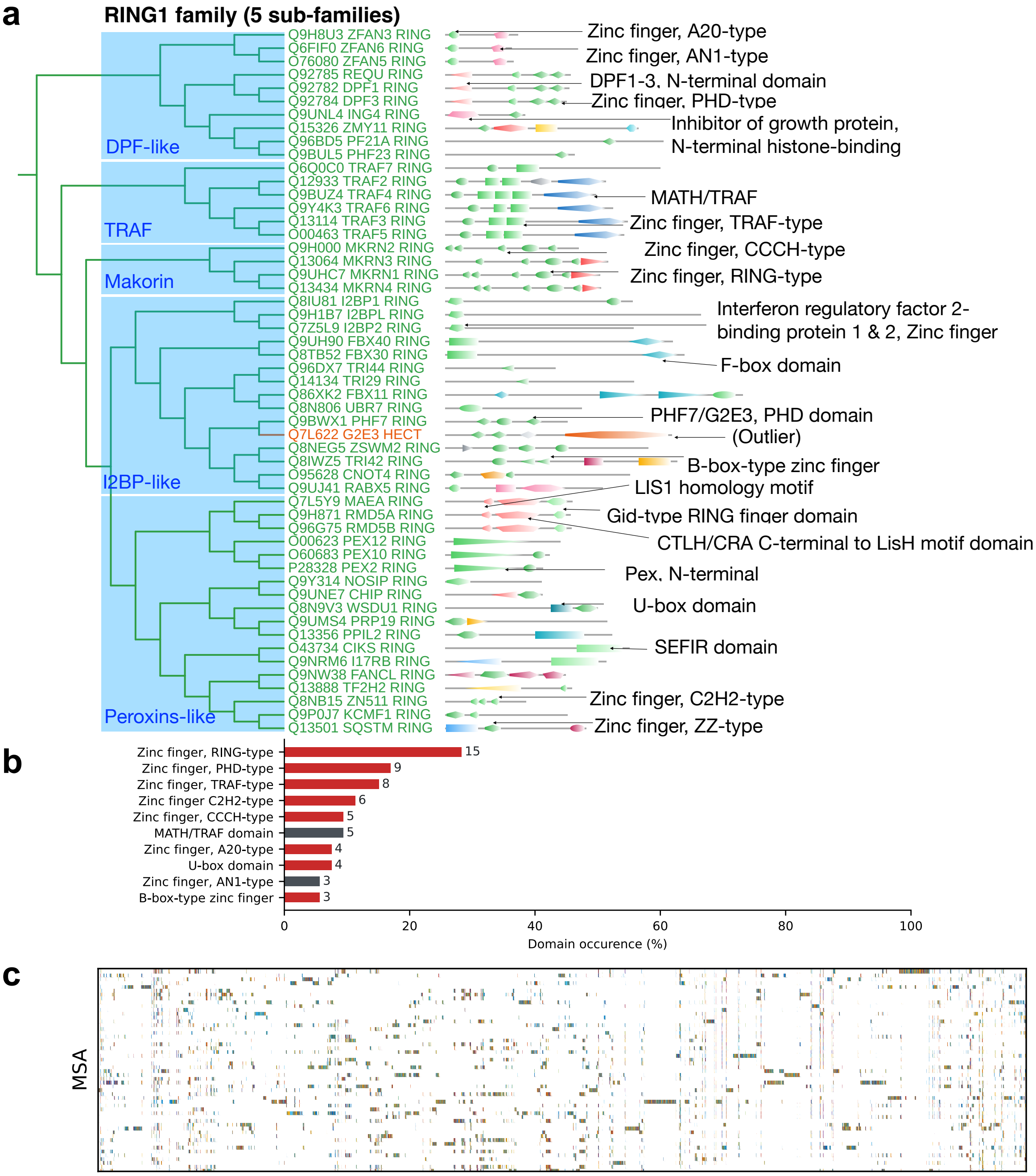

Figure S5: **RING1 family. (a)** Dendrogram showing the organization of 53 E3s corresponding to the RING1 cluster subdivided into 5 subfamilies (labeled). Leaves display individual E3s with schematics of mapped domain architectures. **(b)** Bar plot showing the top 10 enriched domains (catalytic, red; others, black) observed across the cluster. **(c)** A representation of the full-length MSA using T-Coffee with gaps (white) and aligned regions (colored).

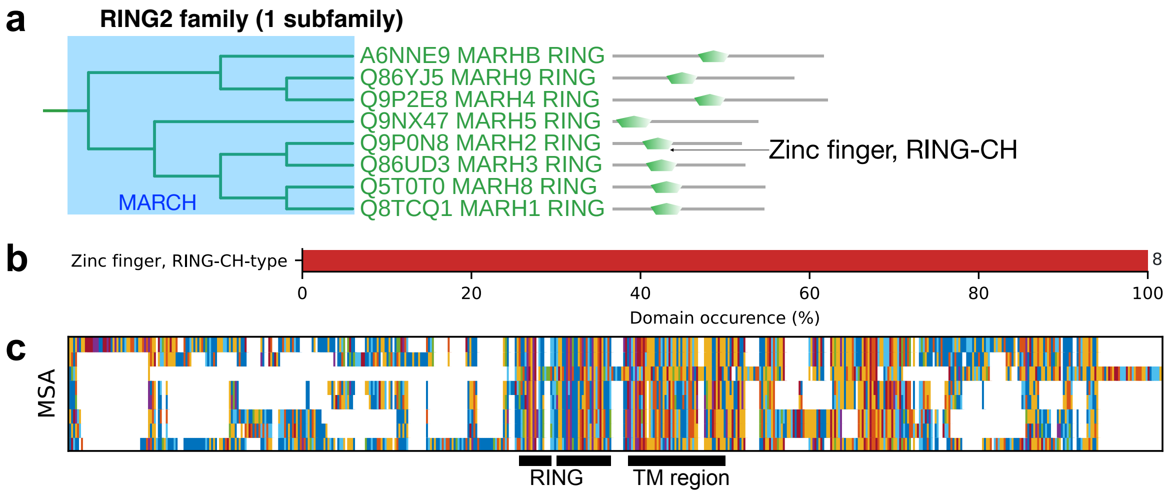

Figure S6: **RING2 family.** **(a)** Dendrogram showing the organization of 8 MARCH E3s corresponding to the RING2 cluster (single subfamily, labeled). Leaves display individual E3s with schematics of mapped domain architectures. **(b)** Bar plot showing the enriched domains (catalytic, red; others, black) observed across the cluster. **(c)** A representation of the full-length MSA using T-Coffee with gaps (white) and aligned regions (colored).

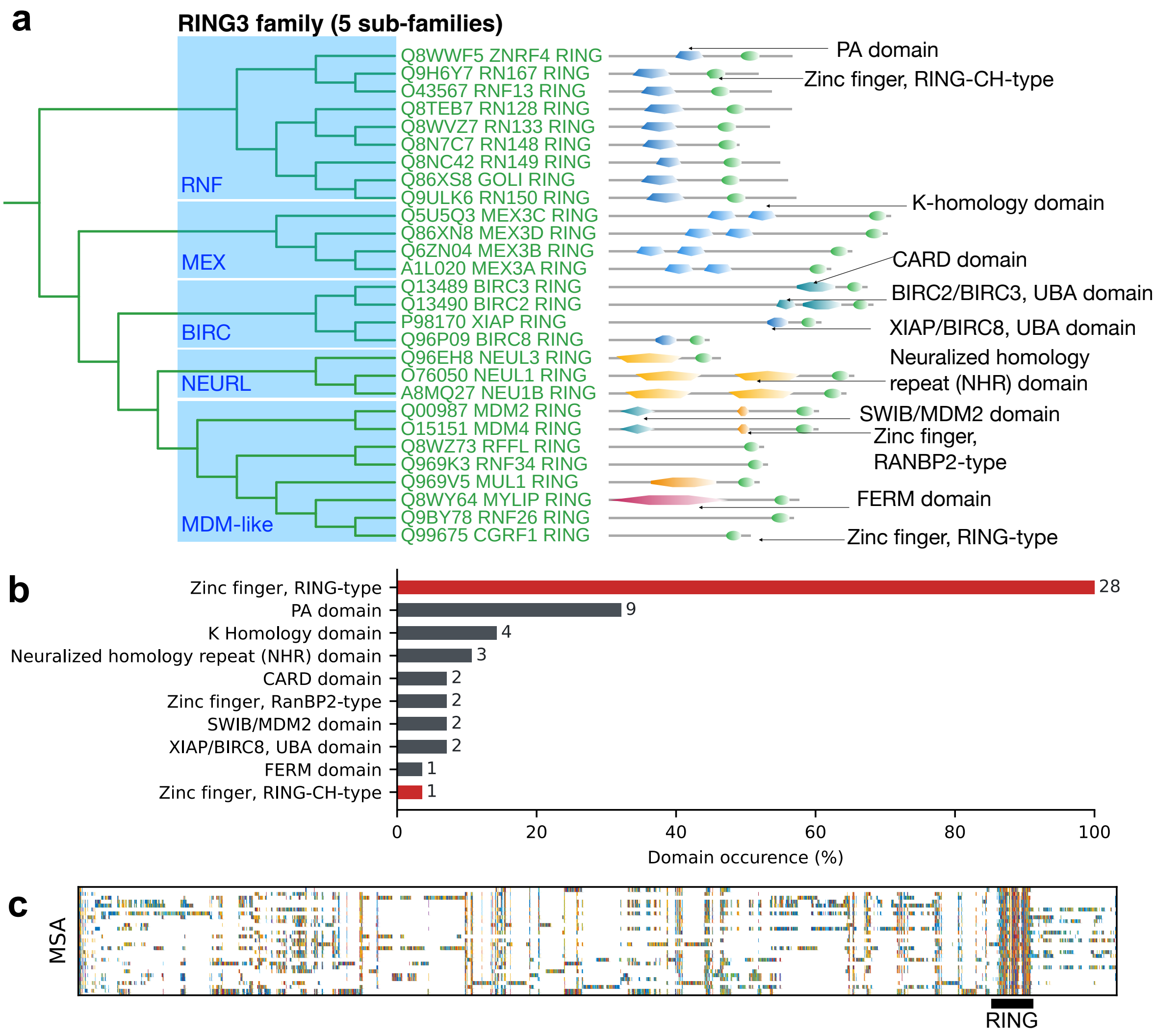

Figure S7: **RING3 family. (a)** Dendrogram showing the organization of 28 E3s corresponding to the RING3 cluster subdivided into 5 subfamilies (labeled). Leaves display individual E3s with schematics of mapped domain architectures. **(b)** Bar plot showing the top 10 enriched domains (catalytic, red; others, black) observed across the cluster. **(c)** A representation of the full-length MSA using T-Coffee with gaps (white) and aligned regions (colored).

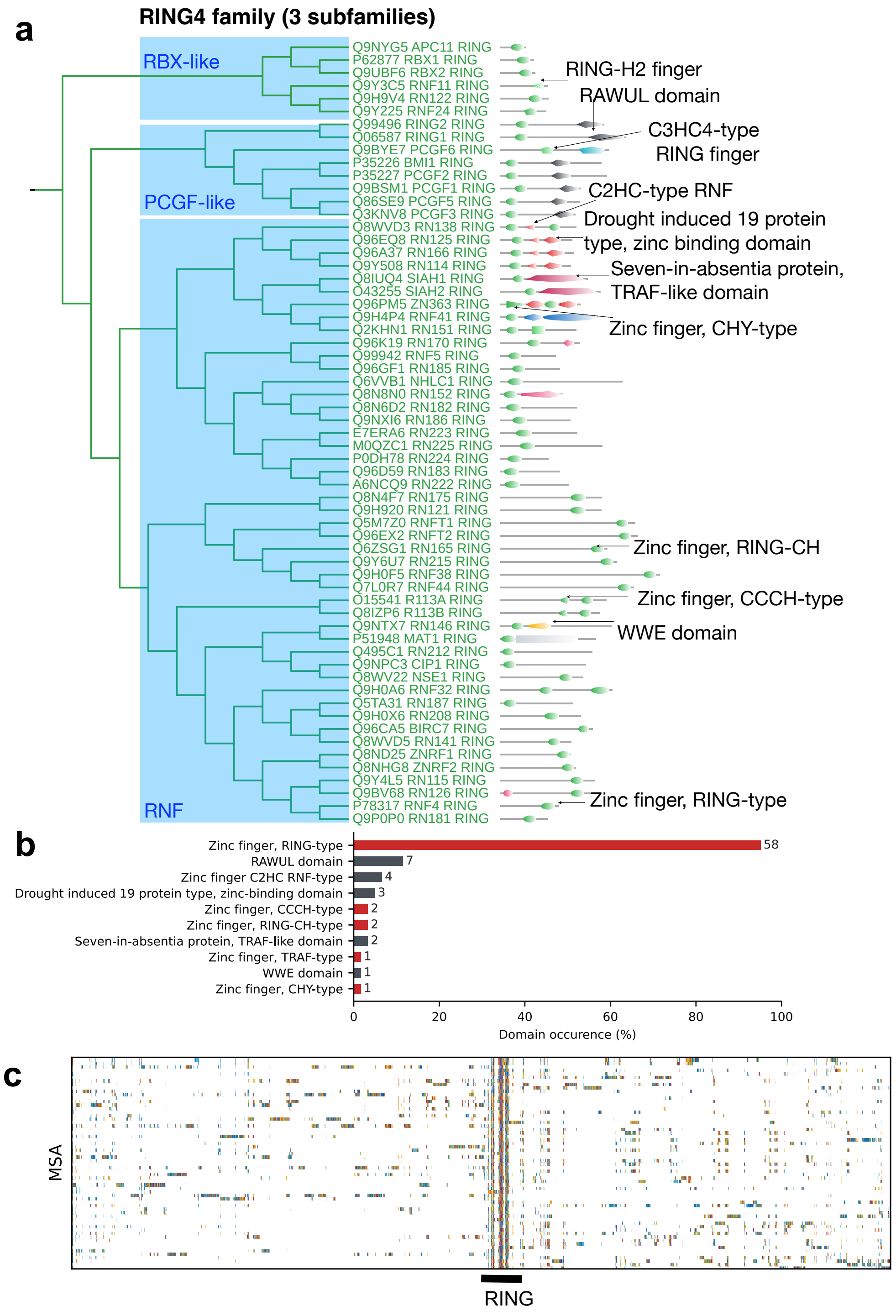

Figure S8: **RING4 family. (a)** Dendrogram showing the organization of 61 E3s corresponding to the RING4 cluster subdivided into 3 subfamilies (labeled). Leaves display individual E3s with schematics of mapped domain architectures. **(b)** Bar plot showing the top 10 enriched domains (catalytic, red; others, black) observed across the cluster. **(c)** A representation of the full-length MSA using T-Coffee with gaps (white) and aligned regions (colored).

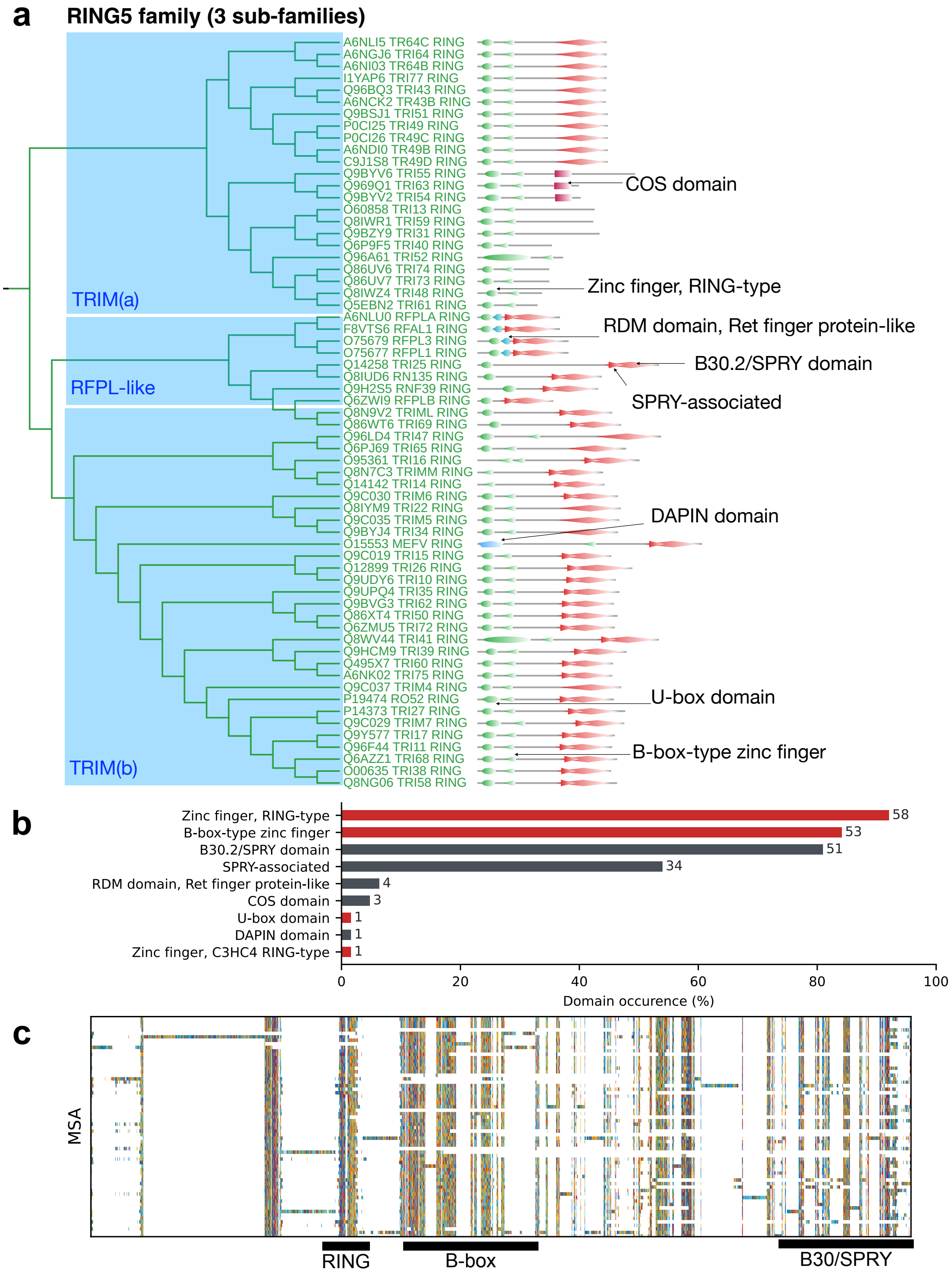

Figure S9: **RING5 family.** **(a)** Dendrogram showing the organization of 63 E3s corresponding to the RING5 cluster subdivided into 3 subfamilies (labeled). Leaves display individual E3s with schematics of mapped domain architectures. **(b)** Bar plot showing the top 10 enriched domains (catalytic, red; others, black) observed across the cluster. **(c)** A representation of the full-length MSA using T-Coffee with gaps (white) and aligned regions (colored).

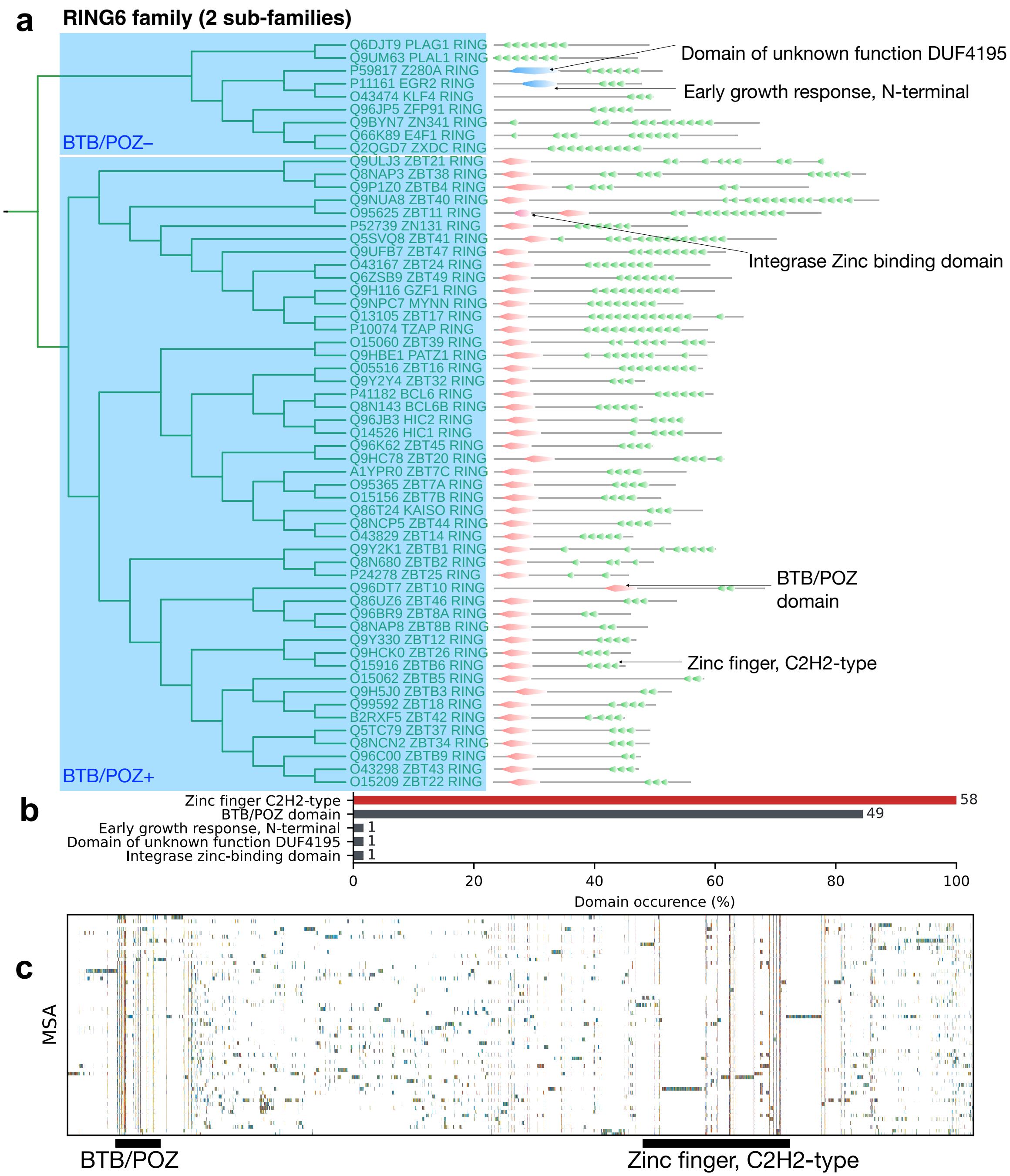

Figure S10: **RING6 family.** **(a)** Dendrogram showing the organization of 58 E3s corresponding to the RING6 cluster subdivided into 2 subfamilies (labeled). Leaves display individual E3s with schematics of mapped domain architectures. **(b)** Bar plot showing the top 5 enriched domains (catalytic, red; others, black) observed across the cluster. **(c)** A representation of the full-length MSA using T-Coffee with gaps (white) and aligned regions (colored).

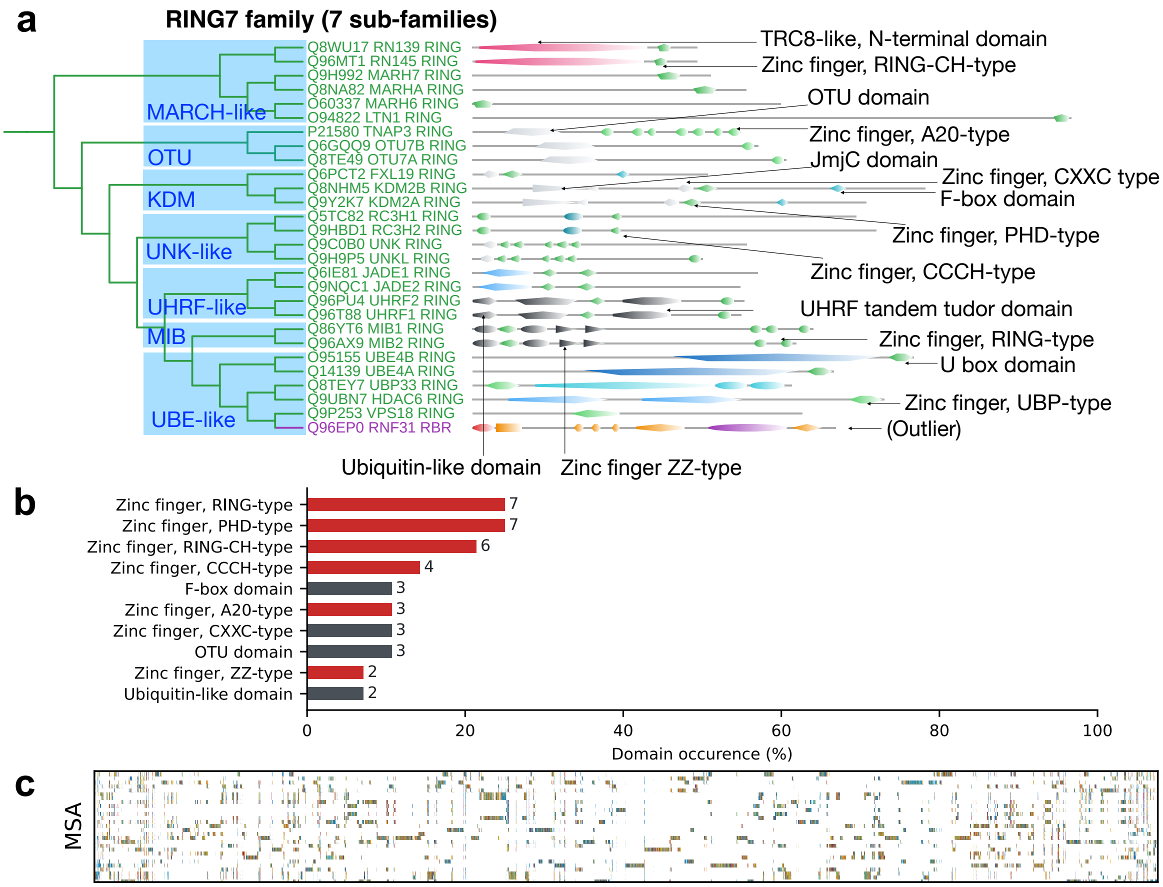

Figure S11: **RING7 family.** **(a)** Dendrogram showing the organization of 28 E3s corresponding to the RING7 cluster subdivided into 7 subfamilies (labeled). Leaves display individual E3s with schematics of mapped domain architectures. **(b)** Bar plot showing the top 10 enriched domains (catalytic, red; others, black) observed across the cluster. **(c)** A representation of the full-length MSA using T-Coffee with gaps (white) and aligned regions (colored).

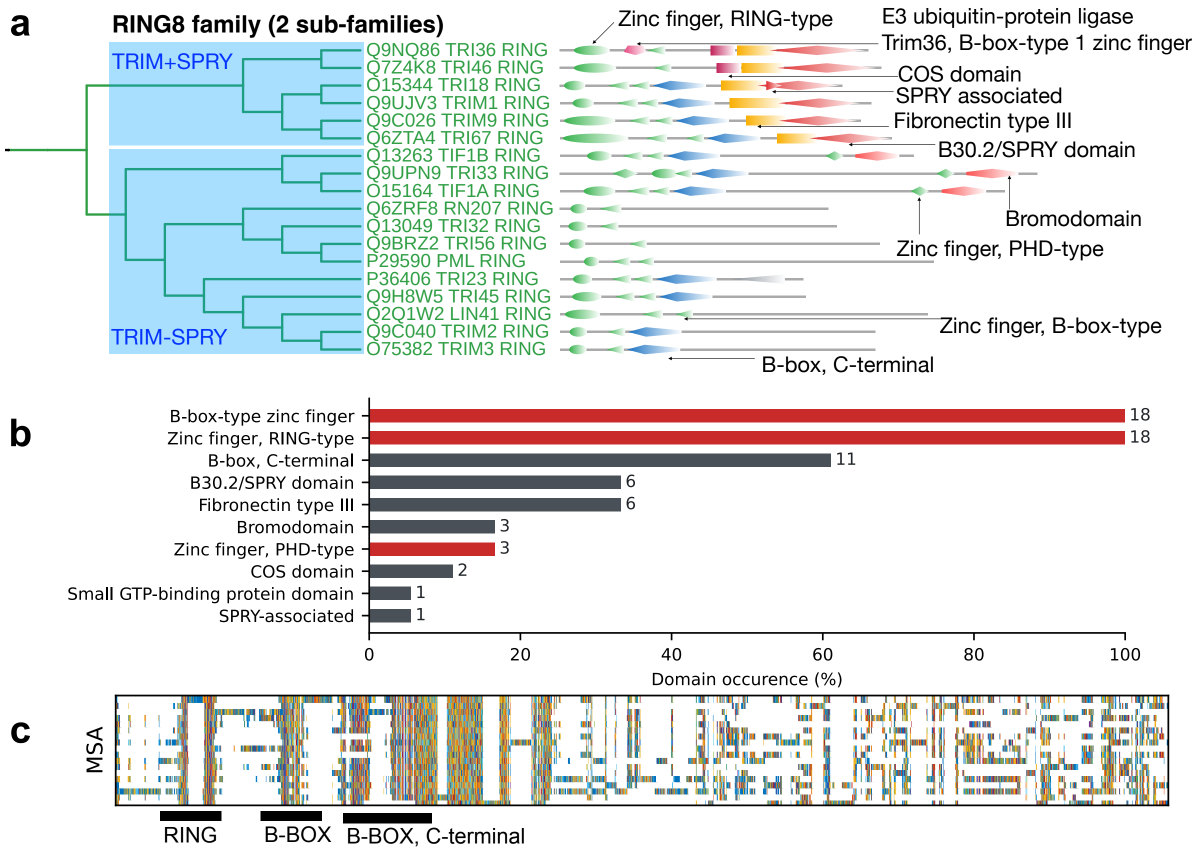

Figure S12: **RING8 family. (a)** Dendrogram showing the organization of 18 E3s corresponding to the RING8 cluster subdivided into 2 subfamilies (labeled). Leaves display individual E3s with schematics of mapped domain architectures. **(b)** Bar plot showing the top 10 enriched domains (catalytic, red; others, black) observed across the cluster. **(c)** A representation of the full-length MSA using T-Coffee with gaps (white) and aligned regions (colored).

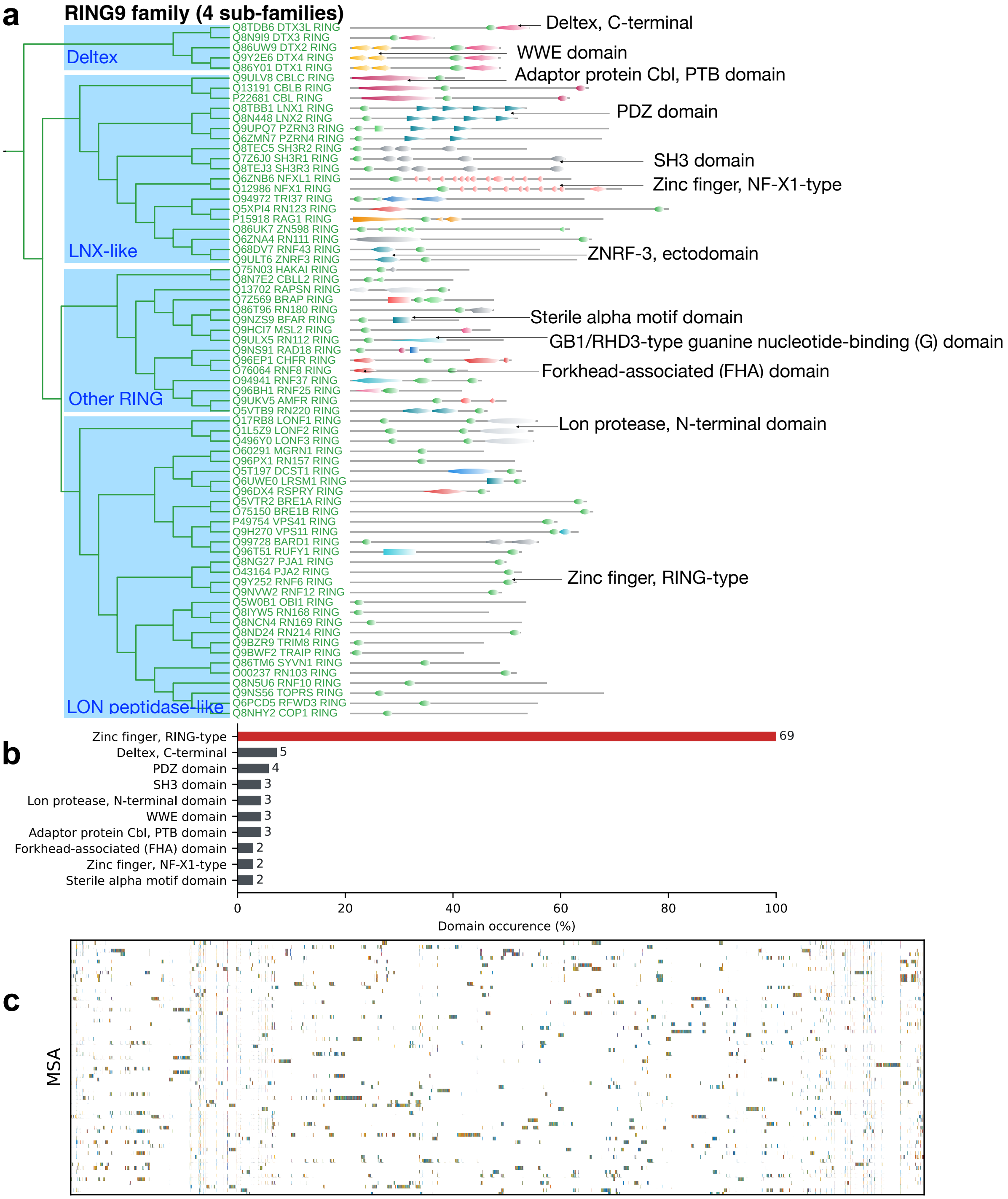

Figure S13: **RING9 family.** **(a)** Dendrogram showing the organization of 69 E3s corresponding to the RING9 cluster subdivided into 4 subfamilies (labeled). Leaves display individual E3s with schematics of mapped domain architectures. **(b)** Bar plot showing the top 10 enriched domains (catalytic, red; others, black) observed across the cluster. **(c)** A representation of the full-length MSA using T-Coffee with gaps (white) and aligned regions (colored).

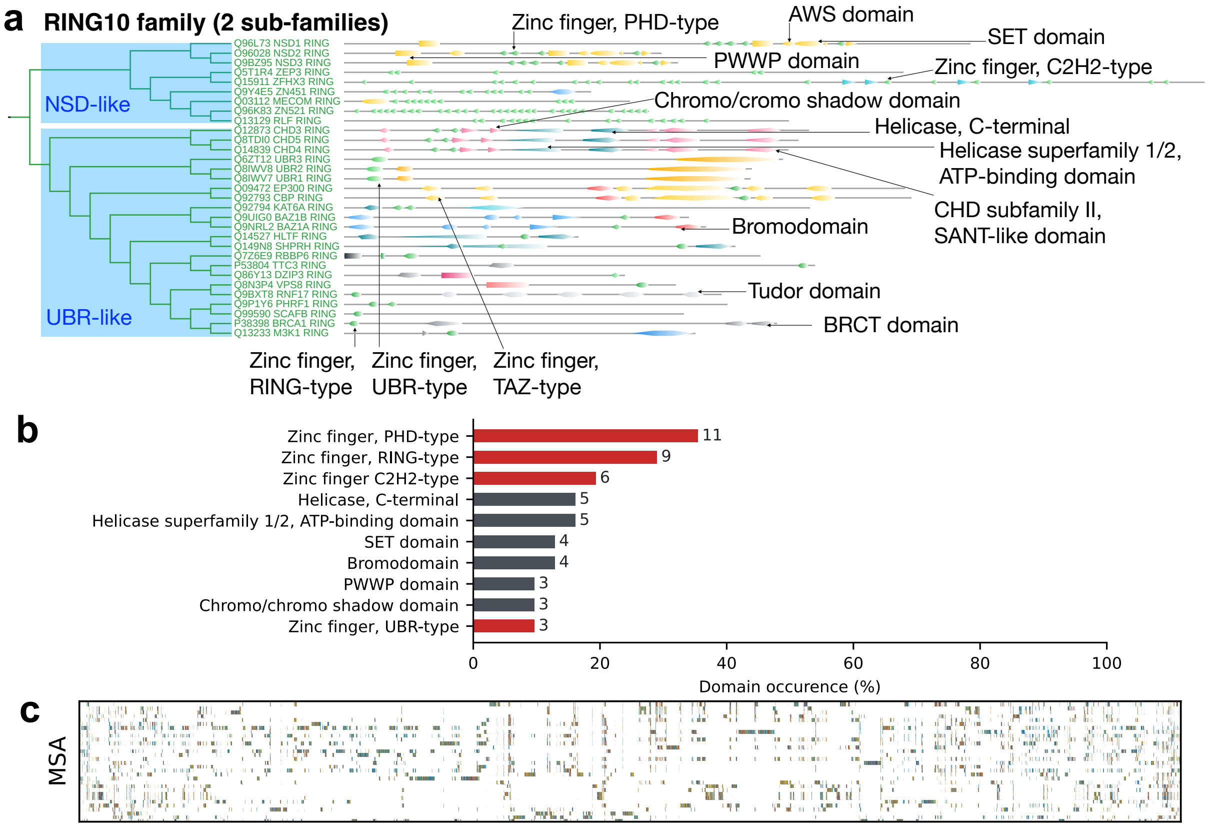

Figure S14: **RING10 family. (a)** Dendrogram showing the organization of 31 E3s corresponding to the RING10 cluster subdivided into 2 subfamilies (labeled). Leaves display individual E3s with schematics of mapped domain architectures. **(b)** Bar plot showing the top 10 enriched domains (catalytic, red; others, black) observed across the cluster. **(c)** A representation of the full-length MSA using T-Coffee with gaps (white) and aligned regions (colored).

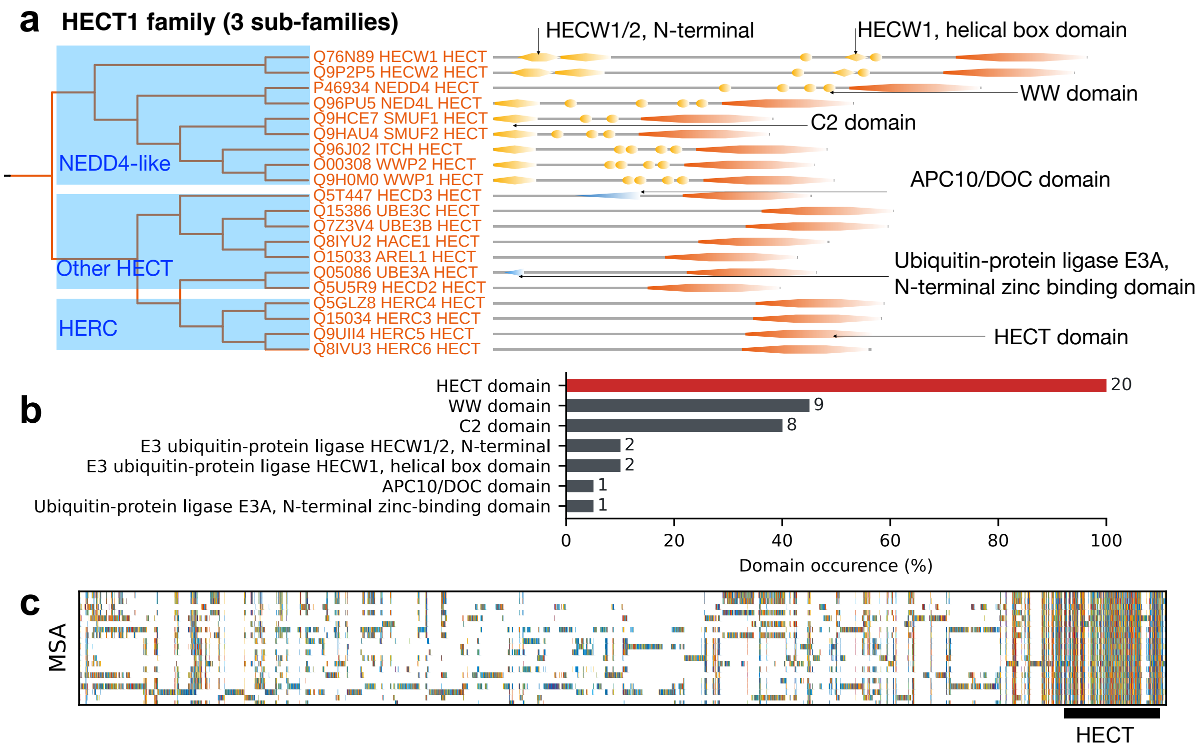

Figure S15: **HECT1 family. (a)** Dendrogram showing the organization of 20 E3s corresponding to the HECT1 cluster subdivided into 3 subfamilies (labeled). Leaves display individual E3s with schematics of mapped domain architectures. **(b)** Bar plot showing the top 7 enriched domains (catalytic, red; others, black) observed across the cluster. **(c)** A representation of the full-length MSA using T-Coffee with gaps (white) and aligned regions (colored).

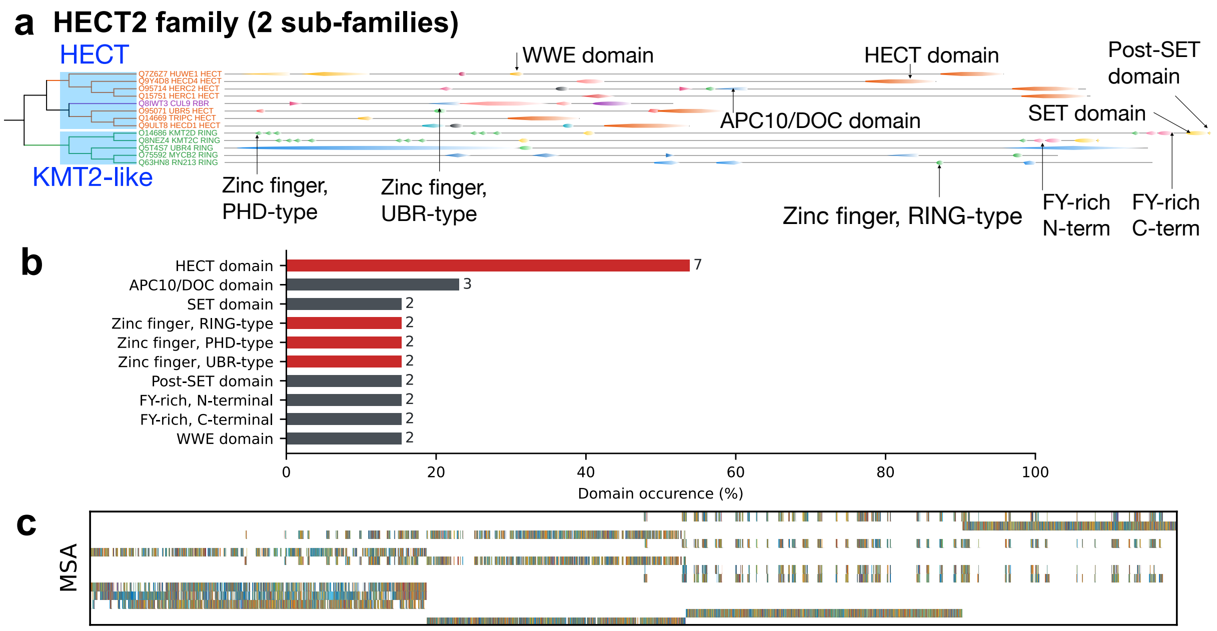

Figure S16: **HECT2 family.** **(a)** Dendrogram showing the organization of 13 E3s corresponding to the HECT2 cluster into 2 subfamilies (labeled). Leaves display individual E3s with schematics of mapped domain architectures. **(b)** Bar plot showing the top 10 enriched domains (catalytic, red; others, black) observed across the cluster. **(c)** A representation of the full-length MSA using T-Coffee with gaps (white) and aligned regions (colored).

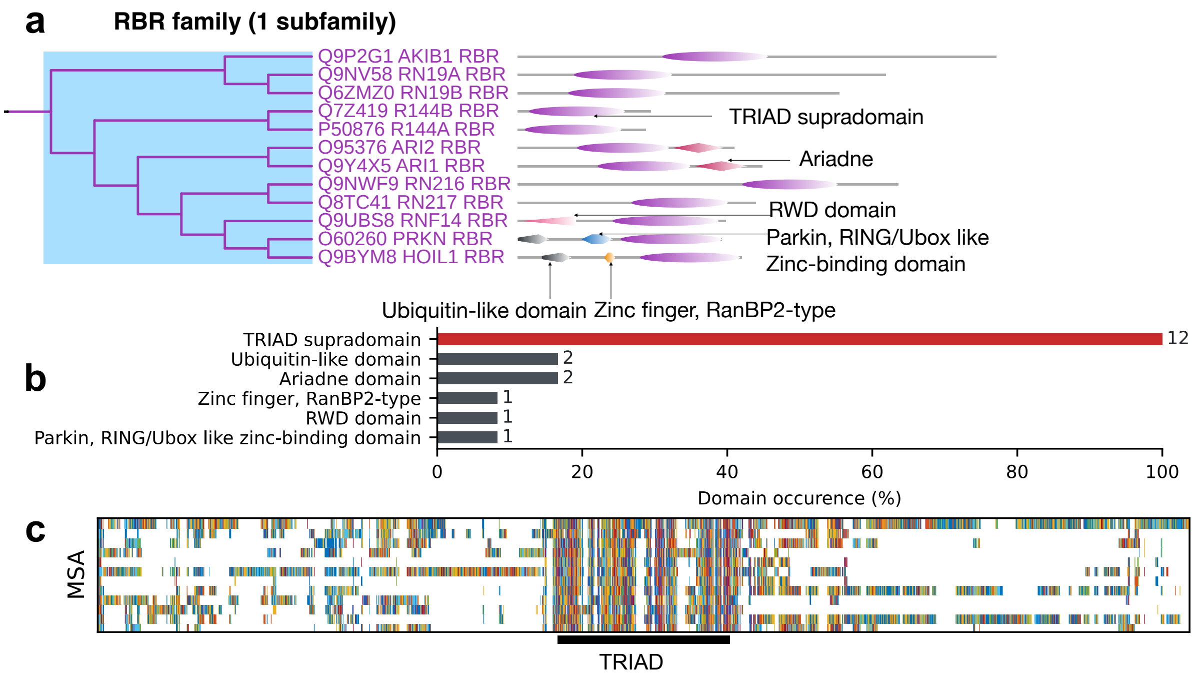

Figure S17: **RBR family. (a)** Dendrogram showing the organization of 12 E3s corresponding to the RBR cluster into a single subfamily (labeled). Leaves display individual E3s with schematics of mapped domain architectures. **(b)** Bar plot showing the top 10 enriched domains (catalytic, red; others, black) observed across the cluster. **(c)** A representation of the full-length MSA using T-Coffee with gaps (white) and aligned regions (colored).

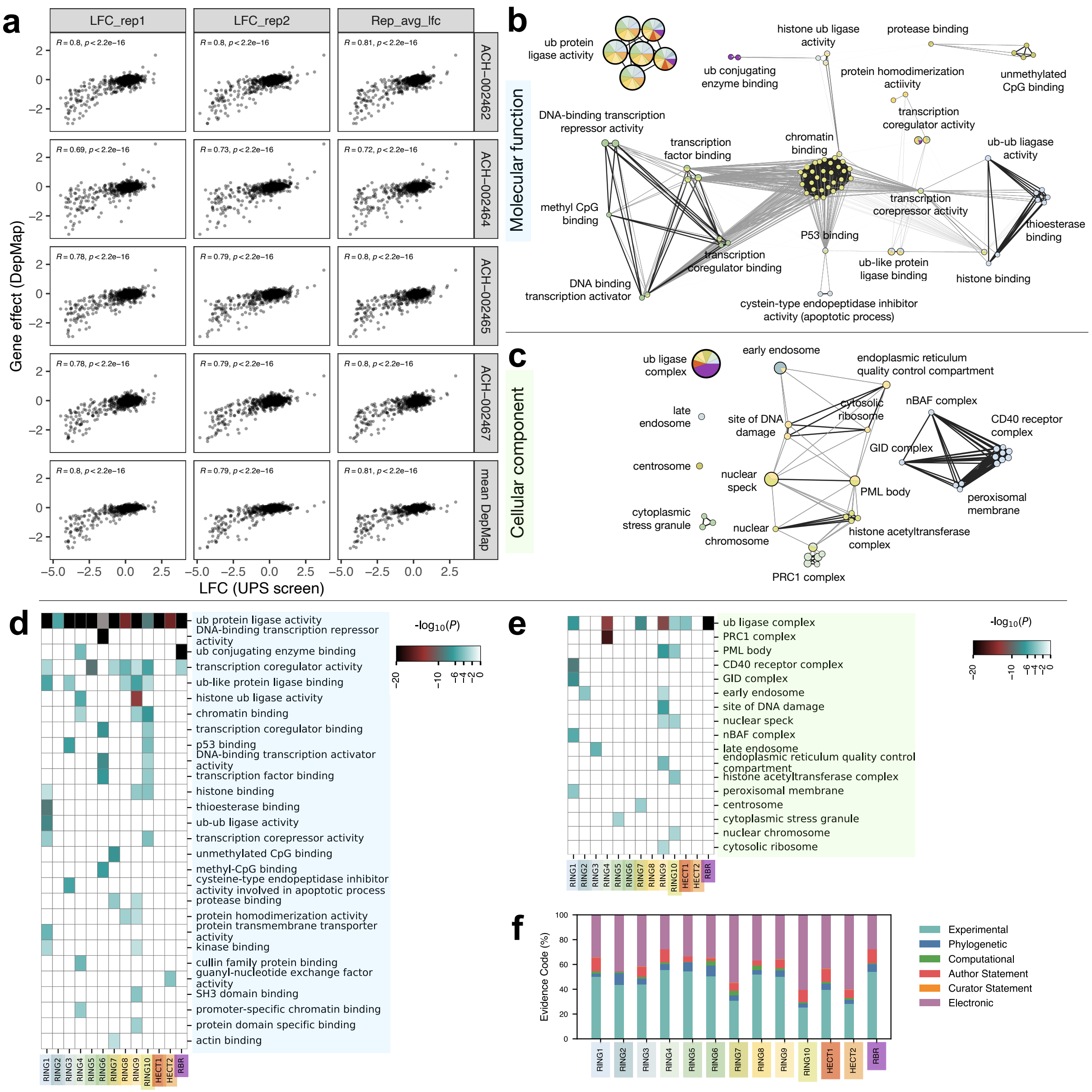

Figure S18: **Molecular function and sub-cellular localization of the E3 ligome.** The functional landscape of the E3 ligome at **(a)** Scatter plots showing the extent of positive correlation (Pearson $r > 0.5$; $n = 822$) between in-house CRISPR screening data (log-fold change for individual replicates and average) with DepMap data (Gene Effect scores). GO enrichment network filtered based on evidence code for top 20 **(b)** molecular function and **(c)** cellular components. The accompanying heatmaps **(d)** and **(e)** display the statistical significance of all enriched clusters using a discrete color scale (Significance threshold p-value cutoff 0.01, in two-sided hypergeometric tests). **(f)** Relative distribution of the GO evidence codes represented as a percentage of all GO terms annotated for each E3 family.

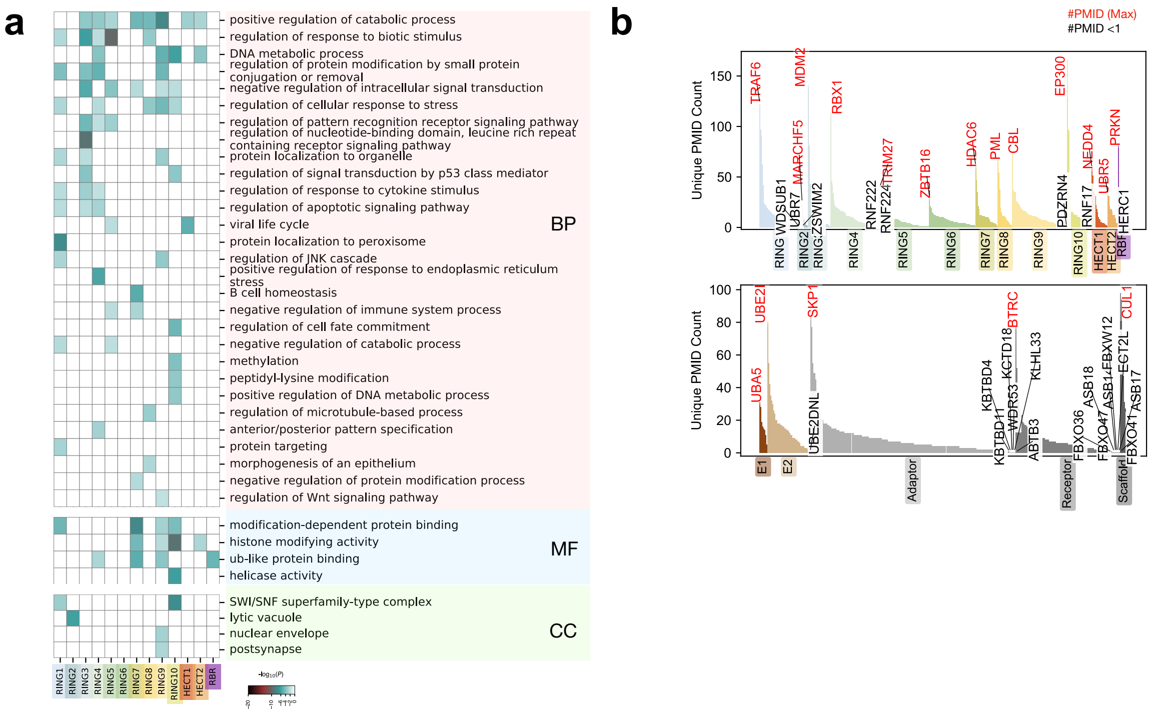

Figure S19: **Predicted functions of the human E3 ligome. (a)** Family-specific GO-enrichment predictions at the biological process, molecular function, and cellular component (GO ontologies) using low-confidence annotations (evidence codes: author statement, curator statement, and electronic annotations) serve as initial hypotheses for future testing. **(b)** Distribution of the unique studies (PMID counts) for each E3 ligase member within the E3 ligome, showing the most extensively studied E3 (red labels), and the least studied E3s (black labels).

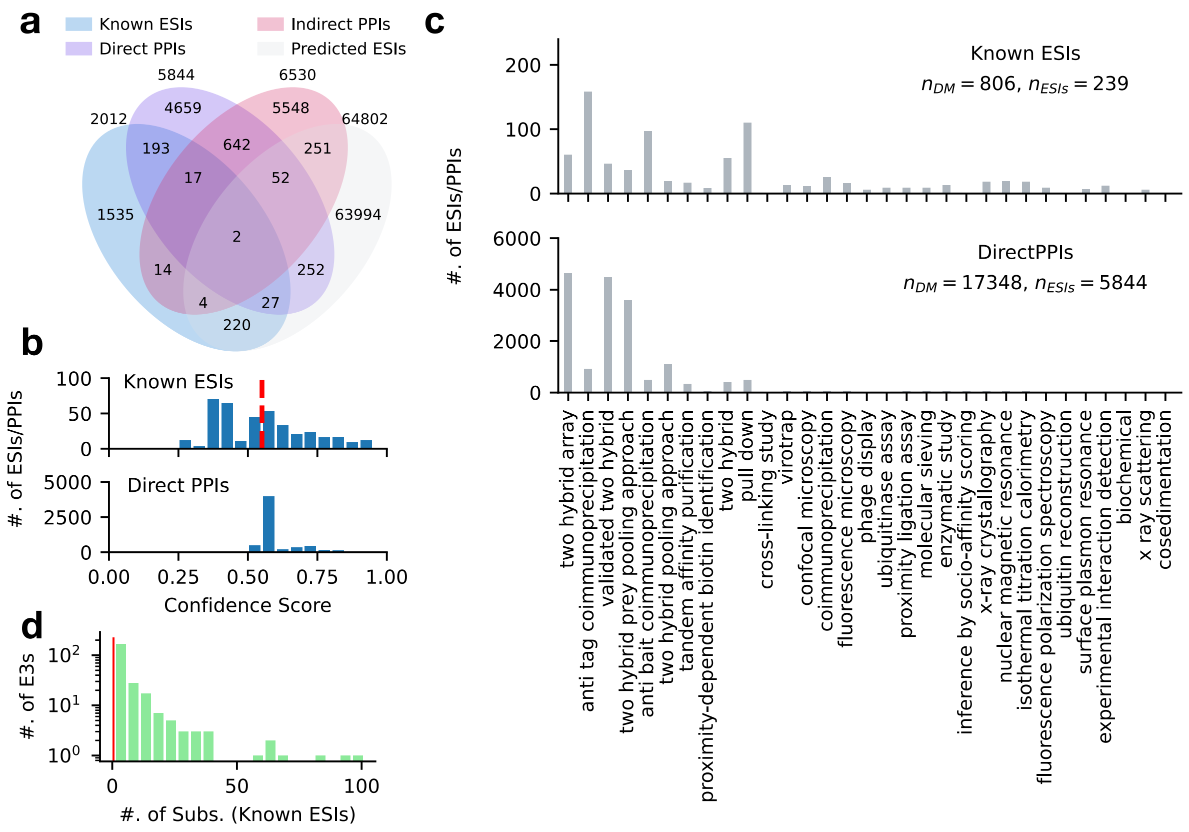

Figure S20: **Mapping ESI networks for the E3 ligome. (a)** Venn diagram showing the overlap of four different datasets used for integrating the complete ESI network of the E3 ligome. **(b)** Histograms displaying the distribution of IntAct MI confidence scores for known ESIs (top) and all PPIs (bottom, IntAct MI confidence scores $\geq$ 0.5). For known ESIs, the IntAct MI confidence scores are available only for overlapping direct PPIs (n = 239; Median value 0.55) and therefore were used to obtain threshold values to filter larger PPI datasets from IntAct. **(c)** Distribution of relative abundance of diverse experimental high-throughput and low-throughput interaction-detection methods used in IntAct MI score estimations, for known ESIs (top) and filtered PPIs (bottom). The total number of detection instances (n_{DM}) corresponding to the total number of interactions (n_{ESIs}) for each group is annotated. **(d)** Histogram showing the distribution of the number of known ESIs per E3 ligase.

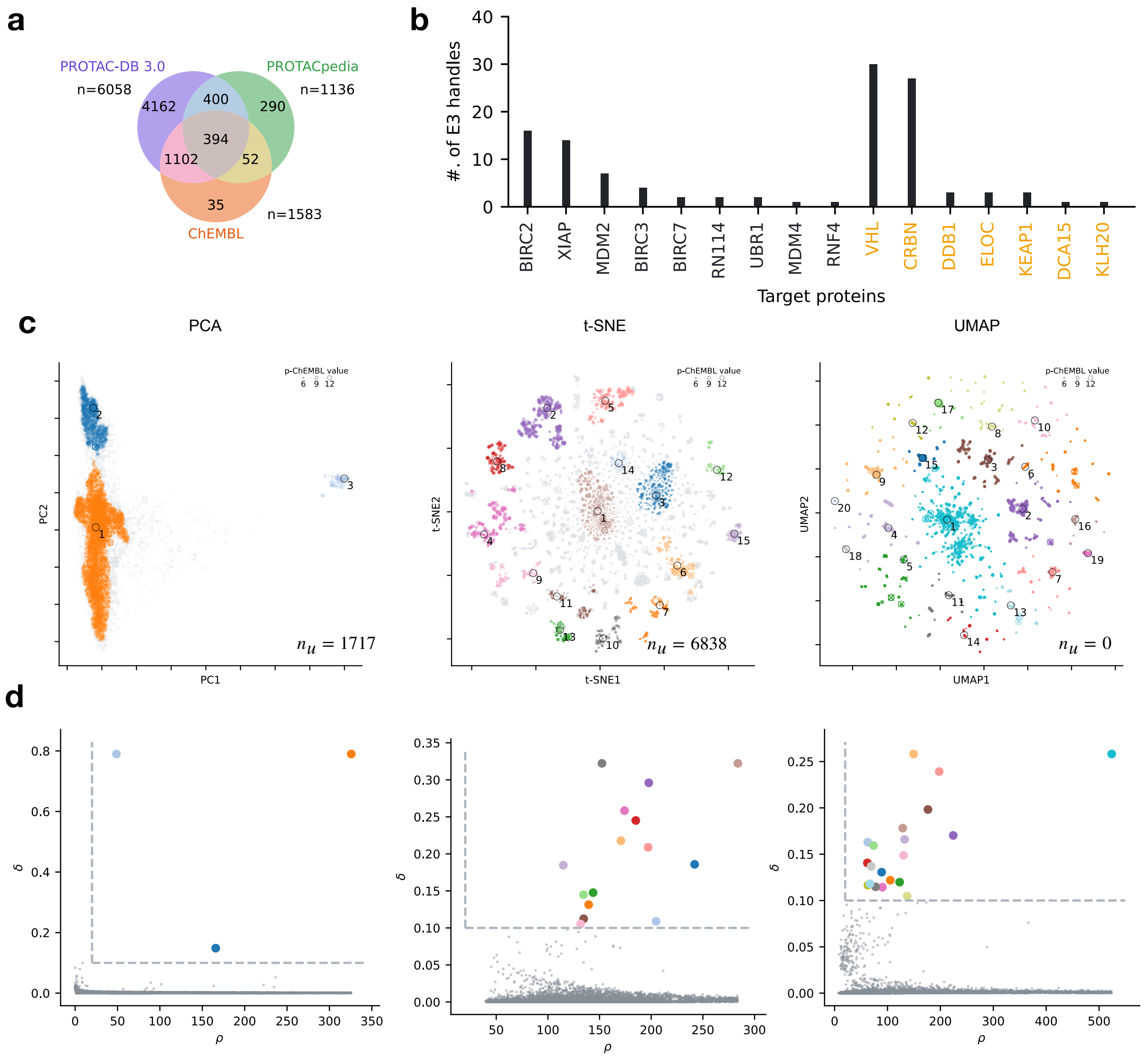

Figure S21: **Chemical space of small molecules targeting E3s. (a)** Venn diagram showing the overlap between PROTACs from PROTAC-DB 3.0, PROTACpedia, and ChEMBL databases. **(b)** Number of unique available E3 handles for each E3 protein target. **(c)** Comparison of dimensionality reduction methods PCA, t-SNE, and UMAP, used to map the chemical space from high-dimensional Morgan (circular) fingerprints of E3 handles and E3 binders (n = 13620; p-ChEMBL ≥ 6.0). Individual clusters (distinct colors) were obtained by finding local density peaks (cluster centers) within corresponding low-dimensional subspaces using **(d)** decision graphs. Local density peaks and corresponding cluster centers are detected with high local density ($\rho_i \geq 20$) and minimal distance to nearest density peak ($\delta_i \geq 0.1$). Note that using PCA and t-SNE subspaces, several molecules remain unassigned (n_u) to density peaks. Only UMAP achieves optimal projection and complete assignment of density peaks, capturing chemical similarities (20 clusters).

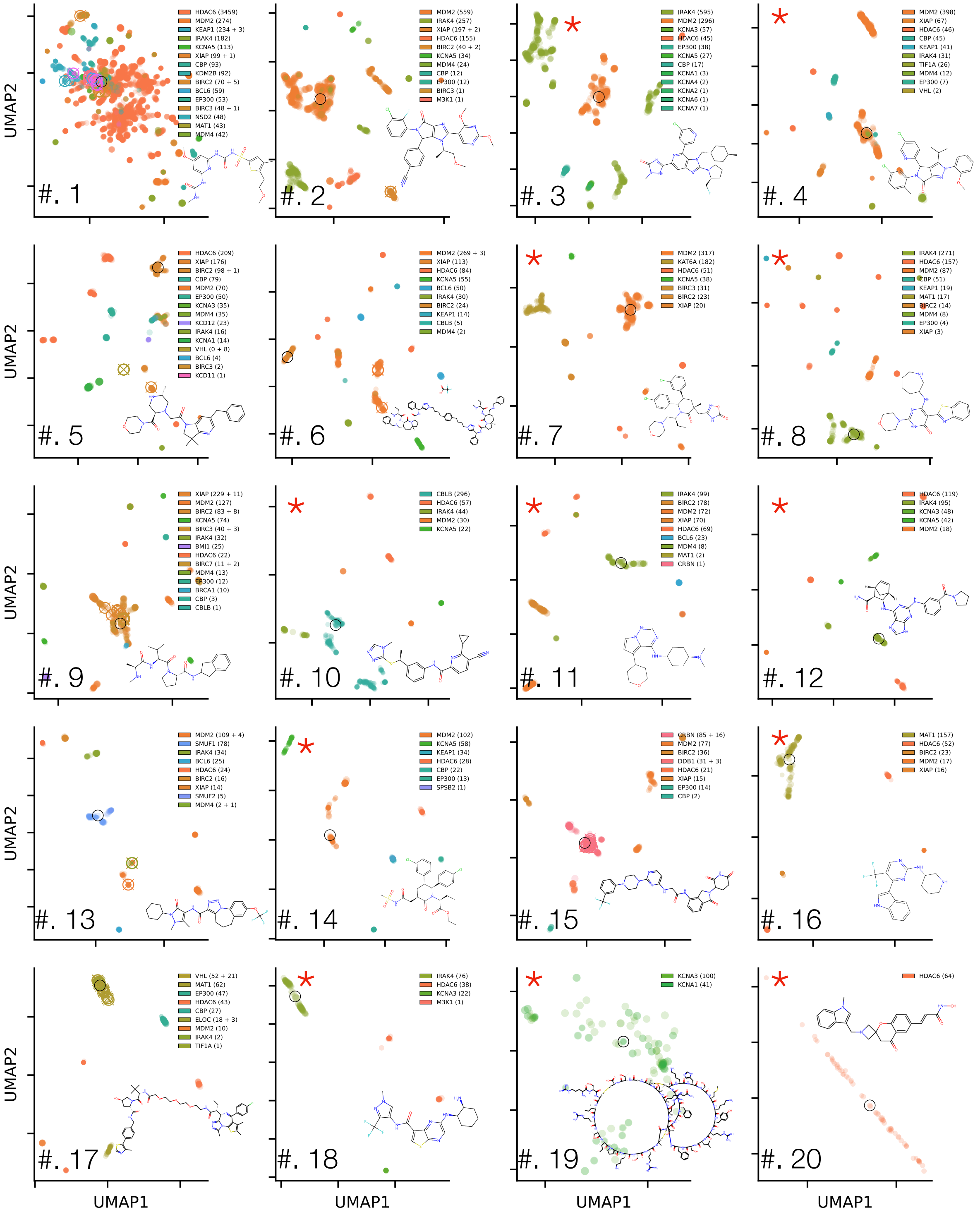

Figure S22: **Cluster characteristics of E3-targeting compounds.** Individual plots show the magnified views along the UMAP sub-space for all 20 clusters. Each cluster is indexed from #1 to #20, by decreasing population and represented by chemical structures (insets, unique chemical scaffolds) corresponding to cluster centers (◯). Individual E3 binders (∘) and handles (⊗) within each cluster are colored based on their corresponding E3 targets and scaled by their p-ChEMBL values. Red * represents clusters with only E3 binders (no E3 handles), useful for developing potential lead compounds for new handle design. Other clusters often contain dense protein-specific sub-clusters proximal to known E3 handles, aiding in drug re-purposing.

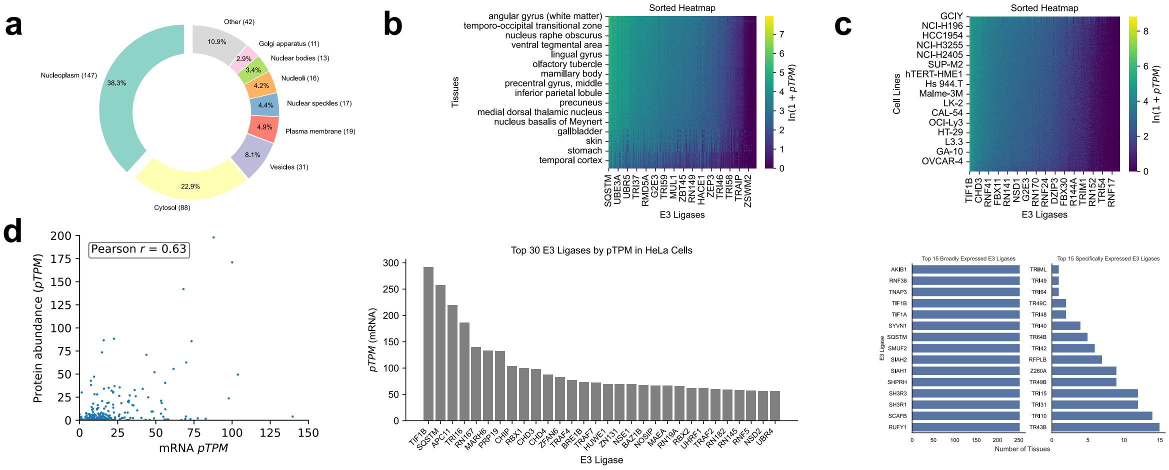

Figure S23: **Projection of E3 distances on systems-level attributes aids effective global drugging strategy.** **(a)** Distribution of the E3 ligome across various subcellular compartments based on primary annotations with approved or supported evidence (n = 298 mapped from Human Protein Atlas). **(b)** Log-transformed pTPM values of 462 E3 ligases across 1055 human cell lines (labeled a few for clarity). **(c)** Log-transformed pTPM values of 462 E3 ligases across 57 human tissues (labeled a few for clarity). **(d)** Correlation between mRNA levels and protein abundance (pTPM values from Human Protein Atlas and PaxDb, respectively) in HeLa cells for E3 ligases (Pearson r = 0.56).**(e)** Expression levels (pTPM) of E3 ligases in HeLa cells indicate the most expressed and least expressed E3s for developing cell-based assays. **(f)** Top 10 most ubiquitously expressed (left) and tissue-specific (right) E3 ligases in humans.

Supplementary Tables

Table S1: **Compilation of the E3 datasets**. The table shows the list of 8 source datasets, their corresponding sizes (the unique number of polypeptides with distinct UniProt IDs, as retrieved in Feb. 2023), the number of E3s with catalytic domains (see **Supplementary Table 2**), and a summary for inclusion criteria.

E3 dataset	Size	Catalytic	Summary
Li et al. (*29*)	601	382	Catalytic and substrate-recognition components of E3 ligase complexes compiled from public databases, including RefSeq, Ensembl, and UniProt.
Deshaies et al. (*9*)	303	299	RING and U-box E3 ligases with demonstrated E3 activity.
Han et al. (*22*)	186	130	E3 ligases with known specificity, extracted by text mining the literature and public databases.
Medvar et al. (*39*)	373	361	RING, HECT, and U-box E3 ligases were compiled based on the presence of catalytic domains.
Liu et al. (*31*)	620	335	Catalytic and non-catalytic components of E3 ligase complexes from Lee et al. (*28*), Li et al. (*29*), and public databases. (Data shared by Prof. Matthieu Schapira.)
Li et al. (*30*)	365	205	E3 ligases compiled from public databases, including E3Net, UbiNet, ESINetwork, and UbiBrowser, while focusing on E3–Substrate interactions.
UniProt (*7*)	1014	294	Extracted with text search “E3 Ubiquitin-protein Ligase” in Feb. 2023.
BioGRID (*45*)	900	421	Themed Curation Project on Ubiquitin–Proteasome system. (Data retrieved in Jan 2022)

Table S2: **Catalytic domains of E3 ligases**. A comprehensive list of catalytic domains featuring in RING, HECT, and RBR classes of E3 ligases was mapped to unique InterPro accessions and used in filtering the catalytic components of E3s from various datasets (also see **Supplementary Note 1** for completeness of the human E3 ligome).

Type	InterPro accession	InterPro description
RING	IPR001878	Zinc finger, CCHC-type
	IPR001841	Zinc finger, RING-type
	IPR008913	Zinc finger, CHY-type
	IPR039515	NOT4, modified RING finger, HC subclass (C4C4-type)
	IPR003613	U-box domain
	IPR011016	Zinc finger, RING-CH-type
	IPR043898	FANCL, UBC-like domain 2
	IPR001607	Zinc finger, UBP-type
	IPR031790	Nitric oxide synthase-interacting protein, zinc-finger
	IPR003126	Zinc finger, UBR-type
	IPR033103	E3 ubiquitin-protein ligase DZIP3, C3H2C3-type RING finger
	IPR006845	Pex, N-terminal
	IPR042981	RING finger protein 11, RING-H2 finger
	IPR007810	Pep3/Vps18/deep orange
	IPR038037	Polycomb group RING finger protein 6, C3HC4-type RING finger
	IPR022750	Interferon regulatory factor 2-binding protein 1 & 2, Zinc finger
	IPR013087	Zinc finger C2H2-type
	IPR000571	Zinc finger, CCCH-type
	IPR001293	Zinc finger, TRAF-type
	IPR002653	Zinc finger, A20-type
	IPR013568	SEFIR domain
	IPR044063	Gid-type RING finger domain
	IPR018957	Zinc finger, C3HC4 RING-type
	IPR000433	Zinc finger, ZZ-type
	IPR000315	B-box-type zinc finger
	IPR001965	Zinc finger, PHD-type
HECT	IPR000569	HECT domain
RBR	IPR044066	TRIAD supradomain

Table S3: Classification of the human E3 ligome. Table summarizing the classification of all 462 human E3 ligases. The table lists all 13 E3 families (class-level assignments), unique subfamilies, representative examples, outliers (class-level), enriched biological processes, cellular components, molecular functions, and previous research (PMIDs).

Table S3:

Family (#)	Subfamily (#)	E.g.	Outliers	Enriched BP	Enriched CC	Enriched MF	Research PMID
RING1 (53)	DPF-like<br>TRAF<br>Makorin<br>I2BP-like<br>Peroxins-like<br>(5)	DPF1<br>TRAF6<br>MKRN1<br>I2BP1<br>PEX2	G2E3 (HECT)	Regulation of NF-κB signaling, K-63 linked ubiquitination, autophagy	CD40 receptor complex, GID complex, nBAF complex	transcription coregulator activity, ubiquitin-ubiquitin ligase activity, protein transmembrane transporter activity	15001576, 10843807, 19536131, 39418515, 33217981, 35463379, 30214450, 35768507, 22113614, 23478441
RING2 (8)	MARCH (1)	MARCH2	–	Protein ubiquitination, polyubiquitination	early endosome	Ubiquitin protein ligase activity	27988304, 31063934, 27577745, 18305173, 37779139, 33653359, 38139010, 17174123, 31404274, 39037545, 31063934
RING3 (28)	RNF<br>MEX<br>BIRC<br>NEURL<br>MDM-like<br>(5)	RN148<br>MEX3A<br>BIRC2<br>NEUL1<br>MDM2	–	regulation of canonical NF-kappaB signal transduction negative regulation of cell cycle, response to hypoxia,	Late endosome	Cys-type endopeptidase inhibitor activity in apoptotic, P53 binding, ubiquitin protein ligase activity	11246007, 17437720, 30095198, 19590513, 35721509, 26232549, 32300648, 38139010, 36647737, 9519875, 18752944, 30525597, 11106166
RING4 (61)	RBX-like<br>PCGF-like<br>RNF<br>(3)	RBX<br>PCGF<br>RNF183	–	ERAD pathway, protein K11-linked ubiquitination, epigenetic regulation of gene expression	PRC1 complex, ubiquitin ligase complex	Cullin family protein binding, histone ubiquitin ligase activity, promoter-specific chromatin binding	31029542, 21103004, 18588675, 11742346, 21610068, 21115485, 32486221, 37760968, 19791798, 32486221

Continued on next page

Table S3: (Continued)

Family (#)	Subfamily (#)	E.g.	Outliers	Enriched BP	Enriched CC	Enriched MF	Research PMID
RING5 (63)	TRIM(a) TRIM(b) RFPL-like (3)	TRIM77, TRIM75, RFPL1	–	Innate immune response, suppression of viral release by the host, regulation of canonical NF-κB signal transduction	Cytoplasmic stress granule	Transcription coregulator activity, ubiquitin-protein ligase activity	38172174, 16498413, 31120853, 28202672, 36266348, 18656177, 25107902, 37770719, 19290053, 21979307, 29949725
RING6 (RING) (58)	BTB /POZ(+) BTB /POZ(−) (2)	ZBT21, PLAL1	–	Hemopoiesis, negative regulation of cell population proliferation, fat cell differentiation	–	DNA binding transcription repressor activity, methyl CpG binding, DNA binding transcription activator activity	29152378, 16207353, 7958847, 7938017, 27250345, 23086144, 34349770
RING7 (28)	MARCH-like OTU KDM UNKL-like UHFR-like MIB UBE-like (7)	RN139 OTU7A KDM2A UNK MIB1 UHRF1	RNF31 (RBR)	ERAD pathway, protein deubiquitination, regulation of cell growth	Centrosome, ubiquitin ligase complex	Actin binding, unmethylated CpG binding, transcription coregulator activity	31404274, 9689122, 16373356, 38195637, 20383180, 38084712, 34016513, 31363749, 34007332, 26170170, 14973063, 18772891, 31481468, 18772889, 38789534, 31462741, 31015428, 39413192, 34016513, 20383180, 38084712, 23334419, 38580649, 29499138, 38172174

Continued on next page

Table S3: (Continued)

Family (#)	Subfamily (#)	E.g.	Outliers	Enriched BP	Enriched CC	Enriched MF	Research PMID
RING8 (18)	TRIM +SPRY TRIM −SPRY (2)	TRIM9 TRIM45	–	Suppression of viral release by the host, innate immune response, positive regulation of autophagy	–	Transcription coregulator activity, ubiquitin-protein ligase activity, protein homodimerization activity	8548820, 24333484, 31120853, 36266348, 28202672, 16498413, 27367845, 25281747
RING9 (69)	Deltex LNX-like LON peptidase Other RING (4)	DTX1 LNX1 LONF1 RNF37	–	Protein K6-linked ubiquitination, response to ionizing radiation, endosomal vesicle fusion	PML body, site of DNA damage, ER quality control compartment	Histone ubiquitin ligase activity, chromatin binding, SH3 domain binding	30295974, 33333989, 11782429, 22889411, 30540932, 24130170, 28129444, 16905136, 9590294, 36197986, 32937373, 39048679, 9204764
RING10 (31)	NSD-like UBR-like (2)	NSD1 UBR1	–	epigenetic regulation of gene expression, protein polyubiquitination, protein acetylation	nuclear speck, histone acetyltransferase complex, PML body	transcription coregulator activity, histone binding, chromatin binding	12372294, 12826405, 12372303, 35532818, 37578463, 39394462, 34722520, 30397230, 29932909, 34789879, 27328812, 39112459, 16311597
HECT1 (20)	NEDD4-like HERC Other HECT (3)	HERC6 NEDD4 UBE3A	–	Regulation of dendrite morphogenesis, regulation of cell growth, protein K-63 linked ubiquitination	Ubiquitin ligase complex	Ubiquitin protein ligase activity	23545411, 19436320, 7846762, 35409239, 29858610, 21947926, 33483704, 35409239, 36918822, 31001145

Continued on next page

Table S3: (Continued)

Family (#)	Subfamily (#)	E.g.	Outliers	Enriched BP	Enriched CC	Enriched MF	Research PMID
HECT2 (13)	HECT KMT2-like (2)	HERC1 RN231	CUL9 (RBR), KMT2D, KMT2C, MYC2B, RN213, UBR4 (5 RING)	Epigenetic regulation gene expression, protein polyubiquitination, protein branched polyubiquitination	–	Ubiquitin protein ligase activity, guanyl-nucleotide exchange factor activity	24722987, 20631078, 33869064, 28193319, 32336296, 38318939, 38453563, 16055722, 39908143, 38182926, 16498413, 30069656, 24793696
RBR (12)	RBR (1)	HHARI PRKN	–	Protein K6 linked ubiquitination, protein polyubiquitination, protein ubiquitination	Ubiquitin ligase complex	Ubiquitin conjugating enzyme binding, ubiquitin-like protein binding, transcription coregulator activity	24576094, 35716664, 36631489, 22420831, 29735995, 32442483, 38605244

Table S4: Literature evidence validating functional segregation of E3 families. Table summarizing the literature evidence (explicit PMIDs) connecting enriched functional terms (GO predictions) with individual E3 ligases from **Fig. 4f-h**.

Table S4:

**Function**	**Enriched E3 ligases**	**PMID**
K6-linked ubiquitination	**RBR**: RNF144A, RNF14, PRKN<br>**RING9**: BARD1, RNF6, RNF8, RNF25	23626584, 25621951, 27863242, 37951215, 37951216, 37955227
Innate immune response (antiviral)	**RING5**: TRIM7, TRIM6, TRIM65, TRIM25, TRIM31, RNF135	14703513, 17392790, 18984593, 28031478, 27992402, 28469175, 31006531, 35263596, 35893676, 9891032
Starvation response (EBSS)	**RING1**: PHF23, TRAF6, SQSTM1, PEX2<br>**RING4**: RNF41, RNF185, RNF166, NHLRC1<br>**RING5**: TRIM13, TRIM21, TRIM27, TRIM22, TRIM65, MEFV<br>**RING8**: MID2, TRIM32, TRIM23<br>**RING9**: DTX3L, CBL, CBLB, CBLC, CBLL1, RNF220, MGRN1, DCST1, LRSAM1, RUFY1, VPS11, VPS41<br>**RING10**: BRCA1, EP300, UBR1, UBR2, UBR3	10362357, 11739384, 11877430, 15258597, 17229889, 17580304, 19363159, 19424180, 19816510, 20168092, 20392859, 20452972, 20534812, 20622874, 21097510, 21148287, 22622177, 23167963, 23486064, 23827681, 24554770, 24790097, 25178484, 25783203, 27498865, 28489822, 31092635, 35670107
DNA damage response (CPT)	**RING1**: ZFAND6, DPF1, DPF2, DPF3, ING4, TRAF2, TRAF3, TRAF4, TRAF5, TRAF6, TRAF7, ZMYND11, ZSWIM2, TRAF3IP2, PRPF19, STUB1, SQSTM1<br>**RING3**: RNF167, ZNRF4, BIRC2, BIRC3, XIAP, MDM2, MUL1<br>**RING4**: BMI1, RNF2, PCGF3, PCGF5<br>**RING5**: TRIM5, TRIM34, TRIM15, TRIM62, TRIM39, TRIM22, TRIM14, TRIM38, TRIM26, TRIM21, TRIM25, TRIM27, TRIM13, TRIM59, TRIM31, TRIM52, TRIM40<br>**RING6**: KLF4, BCL6, ZBTB16, ZBTB17, ZBTB49, ZBTB7B, ZBTB7C<br>**RING7**: RNF139, MARCHF7, TNFAIP3, OTUD7B, KDM2B, RC3H1, UHRF1, UHRF2, MIB2, HDAC6, RNF31<br>**RING9**: BARD1, RLIM, RNF168, TRAIP, TRIM8, RFWD3, COP1, DTX3L, MSL2, RAD18, CHFR, RNF8, RNF25, RNF220, CBL, TRIM37, RAG1<br>**RING10**: BRCA1, EP300, UBR2, CREBBP<br>**HECT2**: TRIP12, UBR5, KMT2D	8702718, 9346484, 9427755, 11053425, 11460167, 11751921, 12296995, 12748188, 12761501, 12857745, 15001576, 15121867, 16314844, 16378096, 17016439, 17135271, 18001824, 18001825, 18079694, 18223652, 18287044, 19160485, 19203578, 19203579, 19500350, 19667203, 19805145, 19818714, 20173098, 20550933, 20651737, 21512573, 21810480, 22607974, 22705371, 22980979, 23077300, 23760478, 23806334, 24413532, 24623306, 25245946, 26474068, 26711499, 27462463, 27667714, 27746020, 27777308, 28073078, 28575657, 28575658, 32094113, 33321094, 33864376

Table S5: Plausible modes of action for the E3 families. List of E3 ligases predicted to operate as part of a complex (based on mapped interactions, Fig. 5d-e), in standalone mode (*31*), or unclassified (not mapped).

Table S5:

Family	Complex E3s	Standalone E3s (*)	Unclassified E3s
RING1	TRAF5, TRAF2, MKRN3, SQSTM, FBX11, TRI42, FBX30, TRI44, RMD5B, TRAF4, RMD5A, CHIP, TRAF6, PPIL2, PRP19, FBX40	NOSIP, ZSWM2, MKRN2, PEX2, I2BPL, PEX12, MKRN1, CNOT4, TRAF7, PEX10, G2E3, FANCL, WSDU1, UBR7, TRI29, TRAF3, PHF7	RABX5, I2BP2, DPF1, KCMF1, ZFAN3, ZFAN6, PF21A, ZFAN5, I17RB, CIKS, MAEA, ZMY11, ING4, ZN511, MKRN4, DPF3, TF2H2, REQU, PHF23, I2BP1
RING2	–	MARH1, MARH5, MARH8, MARHB, MARH2, MARH9, MARH4, MARH3	–
RING3	MDM2, MYLIP	RN150, ZNRF4, RNF13, BIRC8, RN148, RN128, MUL1, RNF26, NEUL1, CGRF1, MEX3C, RFFL, RN167, BIRC3, BIRC2, RN149, NEUL3, MEX3B, XIAP, GOLI, MDM4, RN133, MEX3D, RNF34, MEX3A	NEU1B
RING4	RBX1, RNF4, RING1, ZN363, RING2, PCGF1, RNF32, RNF41, CIP1, APC11, RBX2, RNF11, R113A	RN125, RNF38, RN208, RN151, RN181, RN183, PCGF3, RN212, RN138, RN141, RN121, RN187, RNF24, RN122, RNFT1, BIRC7, NSE1, PCGF2, RN182, R113B, RN152, RN215, RNF5, RN170, MAT1, SIAH1, RN126, ZNRF1, RN185, PCGF6, RN175, RN114, RN115, NHLC1, BMI1, RN166, ZNRF2, PCGF5, SIAH2, RN186, RN146, RN165, RNF44	RNFT2, RN224, RN222, RN223, RN225
RING5	RFPLA, TRI27, RO52, TRI69, TRI50, TRI63, TRI54, TRIM7	TRI41, RNF39, TRI60, TRI61, TRI13, TRI17, TRI35, TRIML, TRI49, TR49B, MEFV, TRI22, TRI64, RFPLB, TRI25, TRI14, TRI34, TRI55, TR49D, TRI77, TRI31, TRI58, TRI15, TRIM6, TRIM5, TRIM4, TRI51, TRI11, TRI10, RN135, TRI48, TRI47, TRI38, TRI62, TRI16, TRI43, TRI40, TRI59, TRI65, TRI73, TRI74, TRI26, TRI68, RFPL1, TRI39, TRI72, TRI52, RFPL3	TRI75, TR64B, TR49C, TR64C, RFAL1, TR43B, TRIMM
RING6	ZBT42, ZBT24, ZBT25, BCL6, ZN131, ZBTB6, ZBT49, KAISO, ZBT34, ZBT44, ZBTB9, ZN341, ZBTB4, ZBT17	–	ZBT10, ZFP91, GZF1, ZBT39, ZXDC, PLAG1, ZBT40, ZBT22, ZBT7A, KLF4, ZBT7B, ZBT14, ZBT21, ZBT43, ZBTB1, ZBT16, ZBT45, ZBT47, PLAL1, ZBTB5, ZBT7C, ZBTB2, HIC2, ZBT26, ZBT8A, Z280A, BCL6B, EGR2, MYNN, ZBT12, E4F1, ZBT37, ZBTB3, HIC1, ZBT32, ZBT8B, ZBT46, ZBT38, ZBT20, ZBT11, TZAP, ZBT41, ZBT18, PATZ1

Continued on next page

Table S5: (Continued)

Family	Complex E3s	Standalone E3s (*)	Unclassified E3s
RING7	FXL19, KDM2B, OTU7A, RN139, UHRF2, UNKL, KDM2A	MARH6, UNK, RC3H1, UBE4A, RC3H2, MIB1, LTN1, MIB2, MARH7, RNF31, RN145, MARHA, UHRF1, UBE4B, VPS18	HDAC6, UBP33, OTU7B, JADE1, TNAP3, JADE2
RING8	TRI18, TRIM3, PML, TRI23, TRI32, TRIM9, TRIM2, TRI36, TRIM1	RN207, TRI46, TRI67, TIF1B, TRI45, TIF1A, TRI33, TRI56	LIN41
RING9	TRI37, LONF1, COP1, LNX1, RNF25	VPS41, RNF12, DTX3, RAPSN, RNF10, CBLL2, BRAP, CBLB, RN157, BARD1, PJA2, RN169, SH3R1, CHFR, RN214, BRE1A, CBL, RN180, RNF43, LONF3, DTX3L, AMFR, SYVN1, RAG1, RN123, DCST1, DTX2, OBI1, RN168, BRE1B, ZNRF3, PZRN3, MSL2, RAD18, TOPRS, RNF6, CBLC, RN220, PJA1, SH3R2, VPS11, TRAIP, PZRN4, ZN598, RNF8, DTX4, TRIM8, DTX1, LRSM1, MGRN1, LNX2, LONF2, RNF37, RN112, RSPRY, RFWD3, RN111, RN103, BFAR, HAKAI	RUFY1, NFX1, NFXL1, SH3R3
RING10	EP300, M3K1, DZIP3	RBBP6, UBR2, SHPRH, RNF17, PHRF1, TTC3, VPS8, UBR3, HLTF, UBR1, BRCA1	CHD5, SCAFB, CHD3, KAT6A, NSD2, MECOM, BAZ1A, ZN451, ZFHX3, NSD1, BAZ1B, NSD3, ZN521, CHD4, ZEP3, RLF, CBP
HECT1	WWP2, NEDD4, UBE3A, HECW1, ITCH, NED4L, WWP1, SMUF2, SMUF1, HECW2	HERC3, HECD2, UBE3C, HERC4, HERC6, UBE3B, HECD3, HACE1, HERC5, AREL1	–
HECT2	–	HECD4, UBR5, HECD1, HERC1, MYCB2, TRIPC, RN213, HUWE1, UBR4, HERC2	KMT2D, CUL9, KMT2C
RBR	PRKN, ARI2, ARI1	R144B, RN216, RNF14, R144A, HOIL1, AKIB1, RN19A, RN217, RN19B	–

Table S6: **Substrate specificity of E3 families.** Summary of the mapped ESI network for each E3 ligase family and their decomposition into aggregated E3-specific, family-specific, and promiscuous ESIs. The number of unique E3 ligases and substrate proteins for each category is also mentioned.

E3 family	Total # of ESI	E3-specific ESIs # ESI (# E3→# Sub.)	Family-specific ESIs # ESI (# E3→# Sub.)	Promiscuous ESIs # ESI (# E3→# Sub.)
RING1	6696	191 (30→191)	50 (13→19)	6455 (53→3055)
RING2	12894	73 (6→73)	2548 (8→554)	10273 (8→2018)
RING3	5913	312 (12→312)	51 (5→25)	5550 (26→3273)
RING4	8373	215 (32→215)	73 (24→34)	8085 (55→3485)
RING5	4170	115 (25→115)	55 (15→21)	4000 (54→1366)
RING6	2940	23 (7→23)	26 (8→7)	2891 (49→1252)
RING7	5905	109 (17→109)	18 (6→9)	5778 (26→2920)
RING8	5158	86 (11→86)	45 (10→20)	5027 (18→2746)
RING9	15324	296 (29→296)	160 (25→66)	14868 (64→4721)
RING10	2515	31 (9→31)	8 (6→4)	2476 (28→1476)
HECT1	18409	270 (9→270)	1949 (11→433)	16190 (20→3273)
HECT2	2107	18 (8→18)	2 (2→1)	2087 (13→1301)
RBR	7162	101 (7→101)	208 (7→65)	6853 (12→3482)

Table S7: Druggability of the E3 ligome. Mapping of E3 handles (extracted from PROTACs) and E3 binders (ChEMBL) for each ligase family, adaptors, receptors, or scaffold proteins. E3-specific, family-specific, and promiscuous compounds are annotated with the target names and the respective number of compounds.

Table S7:

E3 Family	Total handles	E3-spec. handles	Family-spec. handles	Promis. handles	Total binders	E3-spec. binders	Family-spec. binders	Promis. binders
RING1	-	-	-	-	7	-	-	7 [SQSTM (7)]
RING2	-	-	-	-	-	-	-	-
RING3	29	19 [BIRC2 (7), MDM2 (6), XIAP (6)]	8 [BIRC2 (7), BIRC3 (2), MDM2 (1), MDM4 (1), XIAP (6)]	2 [BIRC2 (2), BIRC3 (2), XIAP (2)]	4019	3475 [BIRC2 (119), BIRC3 (10), MDM2 (2697), MDM4 (18), XIAP (631)]	519 [BIRC2 (375), BIRC3 (95), BIRC8 (6), MDM2 (128), MDM4 (128), XIAP (380)]	25 [BIRC2 (11), BIRC3 (17), MDM2 (7), XIAP (8)]
RING4	5	3 [RN114 (2), RNF4 (1)]	-	2 [BIRC7 (2)]	320	299 [BMI1 (25), MAT1 (271), RN114 (3)]	-	21 [BIRC7 (11), MAT1 (10)]
RING5	-	-	-	-	-	-	-	-
RING6	-	-	-	-	161	161 [BCL6 (161)]	-	-
RING7	-	-	-	-	4857	4838 [HDAC6 (4727), KDM2A (21), KDM2B (90)]	2 [KDM2A (2), KDM2B (2)]	17 [HDAC6 (16), RNF31 (1)]
RING8	-	-	-	-	48	31 [TIF1A (29), TRI33 (2)]	14 [TIF1A (14), TRI33 (14)]	3 [TIF1A (3)]
RING9	-	-	-	-	325	325 [CBLB (325)]	-	-

Continued on next page

Table S7: (Continued)

E3 Family	Total handles	E3-spec. handles	Family-spec. handles	Promis. handles	Total binders	E3-spec. binders	Family-spec. binders	Promis. binders
RING10	2	2 [UBR1 (2)]	-	-	717	567 [BAZ1A (1), BRCA1 (10), CBP (209), EP300 (108), KAT6A (182), M3K1 (6), NSD2 (48), NSD3 (3)]	140 [CBP (140), EP300 (140)]	10 [CBP (2), CHD4 (1), EP300 (2), M3K1 (6)]
HECT1	-	-	-	-	80	75 [NEDD4 (2), SMUF1 (73)]	5 [SMUF1 (5), SMUF2 (5)]	-
HECT2	-	-	-	-	-	-	-	-
RBR	-	-	-	-	1	-	-	1 [HOIL1 (1)]
Adaptor	62	55 [CRBN (24), DCA15 (1), KEAP1 (2), KLH20 (1), VHL (27)]	6 [CRBN (3), DDB1 (3), ELOC (3), VHL (3)]	1 [KEAP1 (1)]	3006	2583 [CRBN (59), IRAK4 (1690), KCD11 (1), KCD12 (23), KCNA1 (1), KCNA3 (196), KCNA5 (410), KCNB1 (2), KCNB2 (1), KEAP1 (158), VHL (42)]	159 [CRBN (31), DDB1 (31), ELOC (20), IRAK4 (1), KCNA1 (57), KCNA2 (1), KCNA3 (107), KCNA4 (2), KCNA5 (53), KCNA6 (1), KCNA7 (1), VHL (21)]	264 [CRBN (3), IRAK4 (73), KEAP1 (185), VHL (3)]

Continued on next page

Table S7: (Continued)

E3 Family	Total handles	E3-spec. handles	Family-spec. handles	Promis. handles	Total binders	E3-spec. binders	Family-spec. binders	Promis. binders
Receptor	-	-	-	-	8	8 [SKP2 (5), SPSB2 (3)]	-	-
Scaffold	-	-	-	-	9	9 [CUL4A (9)]	-	-

Supplementary Notes

Note 1: Completeness of the human E3 ligome

To tackle the challenges of data curation, our criterion for inclusion of proteins into the catalytic E3 ligome dataset (n = 462) is objective and considers InterPro domain annotations (Supplementary Table 2) in addition to providing confidence scores based on their presence or absence in various previously cataloged datasets. This exercise showed us that the overlap with previous datasets is poor, and the majority of the proteins from the unified dataset (n = 986) were initially excluded from the catalytic complement of the E3 ligome. Several of these proteins were re-annotated and are categorized into non-catalytic components of the E3 ligome. They constitute E2 proteins (n = 33), scaffolds (n = 8), adaptors (n = 151) and receptors (n = 106) which form multi-subunit E3 ligase complexes. Despite this segregation based auxiliary functions, several proteins remain either unclassified (n = 514) or with no domain annotations (n = 174). Predominantly, these proteins have low-confidence scores and arise from error-prone text-based searches from UniProt and BioGRID entries (Supplementary Table 1). Despite having explicit catalytic domains, several E3s have now been identified as pseudoligases lacking crucial catalytic residues. Few E3s remain untested for explicit catalytic activity or have no information on catalytic functions, while others display minimal activity (currently part of the E3 ligome). The identification and characterization of new E3s and testing their catalytic activity remains an active area of research. A careful analysis of our E3 ligome has enabled us to identify both errors of omission (5 genuine E3s missed) and commission (6 false positives included), discussed below.

Errors of omission

True negatives: Genuine E3s, which could have been missed in the catalytic complement of our E3 ligome, are few. The protein RNF212B, displaying apparent similarity to the RNF sub-family (RING4), is excluded, as there is neither an InterPro domain annotation nor functional characterization to date. Similarly, UBE3D, containing a HECT-like domain sequence, might be mediating both ubiquitination and de-ubiquitination (*40*). Further, PELI1, PELI2, and PELI3 proteins constitute possible omissions in our E3 dataset due to their uncommon C-terminal pellino RING domain (missed in our catalytic domain list).

Errors of commission

False positives: Transcription factors and nucleic acid-binding proteins often get misclassified as E3 ligases owing to their Zn finger domains. For example, CHD3, CHD4, CHD5, and BAZ1A, BAZ1B are chromodomain helicase-DNA-binding and bromodomain-containing transcription factors, respectively, that contain PHD-type zinc finger domains misclassified as E3s (low-confidence). ZNF451 is a SUMO E3 ligase with a zinc finger RING-type domain (misclassified, low confidence score).

Pseudo-E3 ligases

SQSTM1 (RING1) has a zinc finger ZZ-type domain, which is commonly found in authentic E3s, but lacks the conserved residues essential for catalytic function (*3*). It interacts with E2 enzymes UBE2D2/3 and undergoes E2-mediated self-ubiquitination and plays a regulatory role in autophagy pathways (*47*). More recently, a few TRIM proteins characterized by the absence of canonical ubiquitin E3 ligase activity (*13*). This functional deficiency results from structural alterations within the RING domain that affect critical regions involved in either homodimerization or E2 ubiquitin-conjugating enzyme interactions. Current evidence suggests that these pseudoligases may function as regulatory modulators by forming heteromeric complexes with catalytically active E3 ligases, thereby influencing the enzymatic activity of their functional counterparts. Representative members of this pseudoligase group include TRIM6, TRIM15, TRIM22, and TRIM51 (RING5). Similarly, RNF11 (RING4 family), which contains the RING finger H2 domain, modulates other E3 ligases, including SMURF2 and ITCH, and functions as a pseudo-E3 ligase (*38*).

E3s with untested E3 activity or no information

RFPL1 and RFPL4A/B (RING5) lack direct evidence for E3 ligase activity, suggesting they may represent functionally inactive variants. MNAT1 (RING4 family) contains a C3HC4-type zinc finger domain and serves as a subunit of the cyclin-dependent kinase-activating kinase (CAK) complex. However, its inherent ligase activity function remains understudied. Similarly, ZBTB proteins (RING6 family) contain BTB and zinc finger domains and function primarily as transcriptional regulators. Although they are known to form CUL3-based E3 complexes, the direct enzymatic activity remains unclear, with the exceptions of E4F1 and ZFP91 (*24, 27*). Similarly, NSD1, NSD2, and NSD3 are known for histone methyltransferase activity involved in chromatin epigenetic gene regulation, but possess SET domains and zinc finger PHD-type domains (low-confidence).

E3s with minimal ligase activity

MDM4 has been suggested to form heteromeric complexes with MDM2, but itself lacks catalytic activity despite the presence of a RING domain (*42*). E3 ligases VPS11 and VPS41 (RING9), along with VPS8 (RING10), which contain RING domains, likely exhibit reduced ubiquitination activity compared to the E3 ligases VPS18 and VPS11 (*51*). Additionally, other functionally distinct proteins, TNFAIP3, OTUD7A, and OTUD7B (RING7), demonstrate dual roles in ubiquitination and deubiquitination. Their catalytic competence for E3 ligase activity is ultimately determined by active site residues in the A20-type RING domain (*26*). CREBBP and EP300 (RING10) are acetyltransferases with zinc finger ZZ-type domains, associated with E3 ligase function in yeast (*53*), and remain understudied in humans.

Other E3 components

Proteins such as DCAF1, Kelch-type beta propeller domain-containing proteins: KLHDC1, KLHDC2, KLHDC3, KLHDC10 proteins, and FBOX proteins: FBXO21, FBXO43, and FBXO32, which likely function as substrate recognition modules in CRLs, have been included in the non-catalytic E3 receptor family (*12, 50, 60*) after careful analysis (Fig. 1b, Supplementary Figure 1f). Furthermore, many of the unclassified proteins are related to the ubiquitination system–such as members of the MAGE, NEDD8, and SIAH3 families–which are known to associate with E3s, but they neither have an explicit catalytic domain, nor have well-characterized auxiliary roles as adaptor, receptor, and scaffold proteins to data to be classified under the E3 ligome. Until such validation is available, these proteins remain conservatively grouped outside the E3 ligome.

Note 2: Canonical and atypical ubiquitination mechanisms.

Our classification of the E3 ligome broadly reflects well-established catalytic mechanisms—RING, HECT, and RBR (Supplementary Figure 4c). The canonical ubiquitination mechanism leads to the formation of an iso-peptide bond (Schiff's base) between the terminal glycine moiety of ubiquitin and the side-chain lysine of the substrate proteins. RING class of E3s function primarily as scaffolds; they facilitate direct transfer of ubiquitin from the E2 without forming a covalent intermediate, in a conformation that enables efficient ubiquitin transfer. In contrast, HECT E3s carry out a two-step reaction. Ubiquitin is first transferred from the E2 to a catalytic cysteine on the E3, forming a transient thioester intermediate, and then it is transferred from the E3 to the substrate. RBR E3 ligases use a hybrid mechanism. They have two RING domains. RING1 binds the E2 ubiquitin, while RING2 contains a catalytic cysteine that accepts ubiquitin from the E2 via a thioester bond, similar to HECT ligases, before transferring it to the substrate.

Further, our classification also includes non-canonical or atypical E3 ligases. Often, these E3s have classical E3-domains (Supplementary Table S2), grouping them along with the three main classes. For instance, the U-box E3 ligases, such as CHIP (STUB1), are structurally similar to RING-type ligases (see RING1 family; Supplementary Figure 5), but lack zinc-chelating residues, and therefore, rely on hydrogen bonds and salt bridges for their structural integrity and function (*43*). MYCBP2/PHR1 (see heterogeneous HECT2* family; Supplementary Figure 16) employs the RING-Cys-Relay (RCR) mechanism and catalyzes O-linked ubiquitination of serine or threonine residues of substrates (*10, 35*). Similarly, LUBAC (linear ubiquitin chain assembly complex), which includes the catalytic HOIL-1 (RBR family, see Supplementary Figure 17), synthesizes M1-linked linear ubiquitin chains via unique domain interactions (*11*). Another exceptional case is RNF213 (HECT2* family, see Supplementary Figure 16), which ubiquitinates lipid A, a component of bacterial membranes. This process is initiated by attaching a ubiquitin molecule to a hydroxyl group, creating a platform for LUBAC-mediated extension with M1-linked chains (*10*). Further, ARIH1, despite containing the TRIAD supradomain (RBR family, Supplementary Figure 17), uses a trans-thiolation mechanism via a catalytic cysteine, a HECT-like mechanism, making it a hybrid E3 (*4, 48*).

Atypical E3s often (1) form ester bonds (O-linked, or S-linked ubiquitination), targeting non-lysine residues on substrates (e.g., serine, threonine, or cysteine residues). (2) target non-proteinaceous substrates or (3) mediate unique chemical linkages such as phosphoribosylation or (4) display hybrid characteristics. Although such enzymes are not yet widely studied in humans, they exemplify the catalytic flexibility that may exist within the human E3 ligome but remain uncharacterized to date. Furthermore, non-canonical mechanisms are common to host-pathogen systems (*1, 10*). E.g., bacterial (Legionella SidE family) or viral (Herpesvirus MIR1/MIR2) effector proteins mimic human E3s to hijack the ubiquitin machinery, subverting host responses. The presence of atypical E3s within our curated E3 ligome demonstrates the adaptability of our inclusion criteria. The fact that most of these atypical E3s are in the HECT2* heterogeneous family indicates the robustness of our objective metric learning function in capturing alterations in E3 mechanisms (*1, 57*).

Further, there are other well-characterized ubiquitin-like modifier systems in humans (e.g., SUMO, NEDD8, ISG15, FAT10, UFM1, URM1, ATG8/ATG12, etc), each with distinct biological functions (*6*). They mimic the ub-cascade, but often regulate non-proteolytic functions, like sub-cellular localization, protein stability, activity, or interaction of target proteins and other molecules. Although dedicated E3s exist for each of these, only a few, such as SUMO E3s, NEDD8 cascade, and autophagy-related ATG5-ATG12 systems, have been well characterized (*6*). The existence of these distinct E3s indicates shared origins

and subsequent diversification/specialization in multiple lineages from bacteria to humans.

Note 3: RING1 family (DPF-like, TRAF, Makorin, I2BP-like, Peroxins-like)

The RING1 family (Supplementary Figure 5) encompasses 53 RING E3 ligases characterized by one or more catalytic Zinc-finger (Zn-finger) domains, including RING-type (IPR001841), PHD-type (IPR001965), or TRAF-type (IPR001293) domains (Supplementary Figure 5b). This family is organized into five distinct subfamilies (Supplementary Figure 5a). The first subfamily comprises six TNF receptor-associated E3s (TRAFs) that share a unique domain architecture consisting of TRAF-type Zn-finger domains followed by MATH domains. These TRAF ligases are functionally associated with MAPK and JNK signaling pathways, innate immunity, and apoptosis. The second subfamily consists of four Makorin proteins (MKRN1-4), which are putative ribonucleoproteins characterized by a conserved RING-type Zn-finger domain. The remaining three subfamilies, containing diverse E3s with varied domain architectures, are named after their well-known members. The family-level multiple-sequence alignment reveals significant heterogeneity within the DPF-like, I2BP-like, and Peroxin-like subfamilies, as evidenced by numerous gaps (Supplementary Figure 5c). The DPF-like subfamily includes proteins characterized by the DPF domain (double PHD finger proteins, DPF1, DPF3) and members containing tandem repeats of AN20-type and AN1-type Zn-finger domains (ZFAN3, ZFAN5, ZFAN6). The I2BP-like subfamily encompasses proteins from the interferon regulatory factor 2 binding protein (I2BP) family, F-box proteins (FBX30, FBX40, FBX11), and tripartite motif (TRIM) proteins (TRI44 and TRI29), along with UBR7, ZSWM2, RABX5, and the outlier G2E3. Despite its characteristic C-terminal HECT domain, G2E3's presence in this subfamily is explained by its preserved N-terminal domains: an ePHD domain (IPR042012), Ring-type Zn-finger domain, and PHD-PHF7-G2-H3-like (IPR042012) domain, which are also found in the RING E3 ligase PHF7. The Peroxins-like subfamily exhibits diverse domain architectures. Three E3s (PEX2, PEX10, PEX12) with well-preserved Peroxin N-terminal domains are involved in peroxisomal protein import. RMD5A, RMD5B, and MAEA proteins form a distinct subgroup characterized by GID-type Zn-finger and LIS1 homology motifs. The remaining members include the nitric oxide synthase-interacting protein (NOSIP), Cys2His2 Zn-finger proteins (CHIP), and other E3s (CIKS, I17RB, FANCL) containing various Zn-finger domain types (C3HC4-, C2H2-, and RING-type) (Supplementary Figure 5b). The RING1 family demonstrates significant functional segregation (Fig. 4, Supplementary Figure 18), with enrichment in specific biological processes, including regulation of canonical NF-κB signal transduction, K63-linked ubiquitination, and autophagy. At the cellular component level, the family shows enrichment in various complexes, including the CD40 receptor complex, GID complex, and nBAF complex. The family is also associated with molecular functions such as thioesterase binding, ubiquitin-ubiquitin ligase activity, kinase binding, and protein transmembrane transporter activity. The PDB contains approximately 168 structures covering 27 of the 53 RING1 proteins. Within this family, sixteen E3s function as complex E3s, including FBX11, FBX30, and FBX40, which are known to be part of E3 complexes that ubiquitinate BCL6, DTL, and other substrates. Seventeen ligases operate as standalone E3 ligases, and the remaining E3s are currently unclassified (Supplementary Table 5). Analysis of the ESI network for this family revealed 191 unique E3-specific substrates and 19 family-specific substrates (Supplementary Table 6). We identified seven small-molecule binders for the ZZ-type Zn-finger-containing protein, SQSTM (p63) (Fig. 6a), with the potential for the rational design of E3 handles and PROTACs. These molecules could also target other co-clustered E3 within the RING1 family, such as KCMF1.

Note 4: RING2 family (MARCH)

The RING2 family comprises eight membrane-associated RING-CH-type Zn-finger E3 ligases (Supplementary Figure 6). Members of this family are characterized by a catalytic Zn-finger RING-CH type domain (IPR011016) (Supplementary Figure 6a). The RING-CH domain exhibits a distinctive structure compared to the classical RING finger, featuring cysteine and histidine residues at the fourth and fifth zinc-coordinating positions. This RING family is homogeneous and organized into a single subfamily. All the members share a typical structural organization, featuring an N-terminal cytosolic catalytic RING-CH type domain followed by a transmembrane (TM) region containing two TM helices (Supplementary Figure 6b), except for MARCH 5 (contains 4 TM helices). The MSA for this family reveals conservation of both the N-terminal RING domain and TM regions (Supplementary Figure 6c). The longer MARCH E3 ligases ($>$ 700 residues; MARH6, MARH7, MARCH 10) co-cluster with the RING7 family and form a separate subgroup. As integral membrane proteins, these ligases are predominantly localized to endosomes, lysosomes, and the plasma membrane (Fig. 4, Supplementary Figure 18), where they regulate membrane protein ubiquitination and degradation. MARH1 regulates insulin receptor signaling and mediates lysosome-dependent degradation of MHC class II molecules. The PDB resource provides one structure covering 1 out of 8 RING2 proteins. RING2 family members also predominantly function as standalone E3 ligases (Supplementary Table 5). Analysis of the ESI network for this family revealed 73 unique E3-specific substrates and 554 family-specific substrates (Supplementary Table 6). No small molecules or E3 binders are found to target the MARCH family.

Note 5: RING3 family (RNF, MEX, BIRC, NEURL, MDM-like)

The RING3 family comprises 28 RING ligases organized into five distinct subfamilies (Supplementary Figure 7). This family is characterized by a prominent C-terminal Zn-finger RING-type catalytic domain. The multiple sequence alignment (MSA) of the RING3 family demonstrates the conservation of the RING domain across all E3 ligases in this family (Figs. S7b, S7c). The first subfamily consists of nine Ring finger (RNF) ligases that contain the protease-associated (PA) domain (IPR003137), forming a characteristic lid-like structure involved in protein-protein interactions. The four MEX ligases cluster into a separate subfamily, distinguished by the presence of an N-terminal RNA-binding K-homology domain (IPR004087). The third subfamily encompasses four BIRC ligases that contain the baculoviral IAP repeat (BIR) domain. A notable exception among the BIRC proteins is BIRC7, which lacks the BIR1/2 domain and co-clusters with the RING4 family. XIAP (BIRC4) plays a crucial regulatory role in programmed cell death within this subfamily. Another smaller subfamily contains Neuralized-like (NEURL) ligases featuring the Neuralized homology repeat (NHR) domain (IPR006573), which facilitates development in the central and peripheral nervous systems. The last subfamily includes the oncoprotein MDM2 and its homologs (MDM4), along with other proteins possessing a C-terminal RING-type domain (RFFL, RNF34, MUL1, MYLIP, RNF26, and CGRF). The RING3 family demonstrates significant functional segregation (Fig. 4, Supplementary Figure 18). It is strongly associated with signal transduction, negative regulation of the cell cycle, response to hypoxia, and regulation of canonical NF-κB signal transduction. RING3 proteins are primarily associated with the late endosome at the cellular component level. They are enriched in molecular functions such as Cys-type endopeptidase inhibitor activity in the apoptotic process, p53 binding, and ubiquitin ligase activity. The PDB contains approximately 291 structures covering 14 out of 28 RING3 proteins. Most proteins in the RING3 family are likely to function as standalone E3 ligases, while only two proteins are presumed to operate as part of a complex (Supplementary Table 5). Analysis of the ESI network for this family revealed 312 unique E3-specific substrates and 25 family-specific substrates (Supplementary Table 6). MDM2, MDM4, XIAP, BIRC2, BIRC3, and BIRC8 extensively targeted by PROTACs and existing E3 binders (Supplementary Figure 21a). The MDM2 binding compounds occupy a large chemical space. Several E3 handles developed are cross-reactive and interact with other family members, indicating similarities in their binding sites.

Note 6: RING4 family (RBX-like, PCGF-like, RNF)

The RING4 family consists of 61 proteins, with more than 50% classified as RING finger proteins (RNF) containing the Zn-finger, RING-type catalytic domain. A notable feature of this family is the clustering of ligases based on the position of their catalytic domains, with those containing C-terminal and N-terminal catalytic domains forming distinct subfamilies (Supplementary Figure 8a). This clustering pattern reflects sequence-level similarity. In total, three subfamilies were identified within this family. The first subfamily comprises small E3 ligases, including RBX proteins (CRLs) and RNF ligases. The second subfamily consists of eight ligases, corresponding to the Polycomb group (PcG) protein family (PCGF). The “PCGF-like” subfamily is characterized by the presence of a C-terminal RAWUL domain (IPR032443), which mediates interactions with Cbx members of the PRC1 (Polycomb Repression Complex 1) and facilitates homodimer formation. The third subfamily contains 47 RNF ligases with diverse domain architectures, including C2HC-type RNF Zn-finger domains, drought-induced 19 protein-type zinc-binding domains, and Zn-finger SIAH-type profiles (IPR013010), and it shows further internal subdivisions. The C-terminal region of SIAH ligases is thought to function as a substrate-and-cofactor-interaction domain (substrate-binding domain, or SBD), facilitating interactions with various proteins. Twelve RNF proteins containing HC/C3H2C3-type RING domains form a distinct subgroup within this subfamily. Some proteins (RN182, RN183, RN186, RN152) also contain transmembrane domains. The remaining RNF proteins are further categorized based on the location of their RING domains: the N-terminal RING domain is observed in MAT1, RN146, and RN212, while the C-terminal RING domain is present in BIRC7, RN41, and RN126. Additionally, proteins such as RN175, RN121, and RNFTs contain transmembrane domains, likely contributing to their localization within the endoplasmic reticulum (ER). The RING4 family exhibits a well-conserved RING-type domain, as demonstrated in the multiple sequence alignment (MSA) (Supplementary Figure 8c), often occurring in combination with diverse non-catalytic domains. Functionally, this family demonstrates significant segregation. Enrichment analysis indicates involvement in biological processes such as the epigenetic regulation of gene expression, protein K11-linked ubiquitination, and the ER-associated degradation (ERAD) pathway. Members of this family are enriched in the PRC1 complex and the ubiquitin ligase complex. Consistent with this, their enriched molecular functions include cullin family protein binding (CRLs), histone ubiquitin ligase activity, and promoter-specific chromatin binding (Fig. 4, Supplementary Figure 18). The PDB contains 149 resolved structures covering 27 out of 61 RING4 proteins. Most RING4 family proteins are predicted to function as standalone ligases. In contrast, only 13 proteins—primarily from the RBX-like and PCGF-like subfamilies—are expected to function as multi-subunit complexes (Supplementary Table 5). Analysis of the ESI network for this family revealed 215 unique E3-specific substrates and 34 family-specific substrates (Supplementary Table 6). Among the small molecules targeting this family of proteins (Fig. 6a), we identified existing PROTACs and E3 handles for RNF4 and RN114, which could be repurposed to target the co-clustered proteins RN181 and RN166, respectively. Similarly, the new E3 binders identified for proteins MAT1, BMI1, and BIRC7 can be repurposed or developed into potential E3 handles for RN146, PCGF2, and RN141.

Note 7: RING5 family (TRIM(a), TRIM(b), RFPL-like)

The RING5 family is a highly homogeneous family containing 63 E3s. Most RING5 members are TRIM E3 ligases (Supplementary Figure 9a). Three subfamilies were observed in this family: two for TRIM ligases and one for RFLP ligases. In this family, the catalytic Zn-finger RING-type or Zn-finger B-box-type (IPR000315) domains are typically found near the N-terminus (Supplementary Figure 9b). More than 80% of the RING5 family features a B30.2/SPRY domain (IPR001870), a known protein-protein interaction domain with three highly conserved motifs (–LDP–, –LDYE–, and –WEVE–). Flanking the SPRY domain at the N-terminus is the SPRY-associated domain (IPR006574). This unique domain architecture is a basis for categorizing TRIM ligases into two distinct subfamilies. The structural organization of the TRIM ligase in the RING5 family reveals a clear dichotomy: one subfamily, designated TRIM(a), features TRIM ligases with a RING-type domain, B-box motif, and B30.2/SPRY domain. These proteins are characterized by a COS domain but notably lack the C-terminal B30.2/SPRY domain. Conversely, TRIM(b) ligases are distinguished by the presence of both SPRY-associated and B30.2/SPRY domains at their C-terminus. The protein Pyrin (MEFV) from the TRIM(b) subfamily shows the presence of a distinct DAPIN domain at the N-terminal. The DAPIN domain is hypothesized to function as an adaptor involved in the coupling between apoptosis and other immune system disorder pathways. The other subfamily, "RFPL-like," contains the RFPL protein and a few TRIM ligases. They show the presence of both the SPRY-associated domain, B30.2/SPRY domain, and the RING domains at the C-terminus. However, they contain an RDM domain in between. Some of these TRIM ligases have been identified as "pseudoligases" (TRIM6, TRIM15, TRIM22, and TRIM51). The RING5 family is highly homogenous for TRIM family proteins and the SPRY domains. The sequence alignment shows the conserved catalytic RING domain, B-box domains, SPRY-associated, and B30.2 SPRY domain (Supplementary Figure 9c). TRIMs are defined by their multimodular structure, including a conserved RING–B-box–coiled-coil (RBCC) domain and variable C-terminal regions. Despite sharing a similar domain architecture, they perform various functions and are involved in antiviral defense and cancer. The RING5 family demonstrates significant functional segregation. It shows enrichment in the biological process, such as innate immune response, suppression of viral release by the host, and regulation of canonical NF-κB signal transduction. Their most enriched cellular component is the cytoplasmic stress granule, and they are involved in molecular functions such as transcription coregulator activity and ubiquitin-protein ligase activity (Fig. 4, Supplementary Figure 18). The PDB contains 61 structures covering 21 out of 63 RING5 proteins. Most RING5 family proteins are likely to function as standalone ligases; only eight are presumed to function as complexes, and seven E3s are unclassified (Supplementary Table 5). Analysis of the ESI network for this family revealed 115 unique E3-specific substrates and 21 family-specific substrates (Supplementary Table 6). No existing PROTACs or E3 binders are identified for RING5 family proteins.

Note 8: RING6 family (BTB/POZ+, BTB/POZ-)

The RING6 family consists of 58 E3s, characterized by tandem repeats of Zn-finger C2H2-type catalytic (IPR013087) domains, often near the C-terminus (Supplementary Figure 10a). This well-studied Zn-finger domain contains two segments with two cysteines and two histidines (C2H2) coordinating the Zn in a tetrahedral geometry. This functional domain recognizes and binds to nucleic acids and proteins. Additionally, more than 80% of them contain the BTB/POZ domain (Broad-Complex, Tramtrack, and Bric á brac; and Poxvirus and Zn-finger; IPR000210; Supplementary Figure 10b). The N-terminal BTB/POZ domain is essential for protein-protein interactions, facilitating the formation of homo- and heterodimers, interactions with transcription factors, and recruitment of CUL3-based E3 complexes. Consequently, at the molecular function level, we observe enrichment for mediation of DNA binding transcription repressor activity, methyl CpG binding, and DNA binding transcription activator activity (Fig. 4, Supplementary Figure 18). The multiple-sequence alignment of the RING6 family shows the conservation of the C-terminal Zn-finger C2H2 domain repeats and the N-terminal BTB/POZ domain (Supplementary Figure 10c). The RING6 family is segregated into two distinct subfamilies based on BTB/POZ domain presence. The subfamily without the BTB/POZ domain (BTB/POZ–) encompasses nine E3s, including PLAG1 and PLAL1, which feature N-terminal C2H2-type Zn-finger domain repeats. Additional members such as Z280A and EGR2 possess N-terminal DUF (Domain of unknown function) and early growth response domains, facilitating homodimerization and nucleic acid binding. Overall, RING6 members are enriched in specific biological processes such as hemopoiesis, negative regulation of cell population proliferation, and differentiation of fat cells. They are involved in molecular functions such as methyl CpG binding, DNA binding, transcription repressor, and activator activity. The PDB contains 172 resolved structures, covering 15/58 RING6 proteins. Fourteen RING6 family proteins are likely to function as complex ligases; however, other proteins remain unclassified (Supplementary Table 5). Analysis of the ESI network for this family revealed 23 unique E3-specific substrates and seven family-specific substrates (Supplementary Table 6). We detected 161 compounds binding to BCL6, clustered into three groups. These represent the potential for the rational design of E3 handles and PROTACs (Fig. 6a).

Note 9: RING7 family (MARCH-like, OTU, KDM, UNKL-like, UHFR-like, MIB, UBE-like)

The RING7 family consists of 27 RING E3s (Supplementary Figure 11a). This family has members with diverse Zn-finger domains (Supplementary Figure 11b), making it heterogeneous. This family is organized into seven distinct subfamilies. The Lysine demethylase (KDM2) ligases subfamily is characterized by a distinctive multi-domain architecture featuring an N-terminal Jumonji C domain, CXXC-type and PHD-type Zn-finger domains, and a C-terminal F-box domain. The ovarian tumor protease (OTU) E3 ligases form a separate subfamily, displaying the OTU and Zn-finger A20-type domains. They function as the deubiquitinase and ubiquitin modification enzymes in the NF-κB pathway. The subfamily of Mind Bomb (MIB) ligase shows the presence of the N-terminal MIB-herc2 domain, a Zn-finger ZZ-type domain, followed by the MIB-herc2 domain, MIB SH3 repeat domains, and C-terminal Zn-finger RING domains. The remaining four subfamilies contain many E3s with varied domain architectures and are named after well-known E3s within these sub-clusters. The "MARCH-like" subfamily contains 3 MARCH E3s and the other RNF proteins. These proteins share a Zn-finger RING-CH type catalytic domain. The three longer MARCH proteins (MARH6, MARH7, and MARHA/MARCH 10) co-cluster within this family of large E3 ligases ($\sim$ 600 residues or longer). MARH7 and MARHA lack transmembrane domains, while MARH6 contains several transmembrane domains orthologous to the yeast E3 ligase Doa10. This subfamily includes the Listerin (LTN1) ligase and two RNF proteins (RN139 and RN145). RN139 and RN145 are characterized by a TRC8-like N-terminal domain, encompassing 12 transmembrane domains and a putative sterol-sensing domain. The "UNK-like" proteins constitute a distinct subfamily along with RNA-binding RC3H (ROQUIN) proteins. The characteristic feature of this subfamily is the presence of a Zn-finger CCCH-type domain. Within this subfamily, members cluster primarily based on domain composition similarity. RC3H proteins display a distinctive domain organization, featuring an N-terminal Zn-finger RING-type domain followed by a Roquin II domain and a C-terminal Zn-finger CCCH-type domain. The UNK-like ligases exhibit a unique architectural arrangement comprising an N-terminal Unkempt Zn-finger domain succeeded by multiple Zn-finger CCCH-type domain repeats. The UHRF and JADE ligases form a separate subfamily ("UHRF-like"), distinguished by their Zn-finger PHD-type domains. UHRF family members possess a more intricate domain architecture, consisting of an N-terminal Ub-like domain, UHRF tandem tudor domain, Zn-finger PHD-type domain, and SRA-YDG domain - the latter mediating nucleic acid interactions - followed by a C-terminal Zn-finger RING-type domain. The functional roles of both the Zn-finger PHD-type and SRA-YDG domains are evidenced by enrichment in molecular functions, particularly histone-modifying activity and unmethylated CpG binding. The JADE ligases feature an N-terminal enhancer of a polycomb-like domain followed by a PHD-type domain. The other heterogeneous subfamily, "UBE-like," contains 5 E3s and RNF31 (outlier, RBR-class). This subfamily shows the presence of different catalytic domains, such as the U-box domain, Zinc finger, UBP-type, and Pep3/Vps18/deep orange. The E3 ligase UBE shows the presence of the Ubiquitin conjugation factor E4, core, and the U-box domain. The UBE proteins are involved in functions related to DNA damage. Other family members, UBP33 and HDAC6, share a UBP-type Zn-finger domain, with UBP33 additionally containing ubiquitin-specific protease (USP) and DUSP domains. HDAC6 is distinguished by a histone deacetylase Zn-finger domain, which facilitates its histone modification function. VPS18 is characterized by a Pep3/Vps18/deep orange domain, which mediates trans-Golgi network trafficking. The heterogeneity of these subfamilies is reflected in the family-level multiple sequence alignment, which exhibits numerous gaps (Supplementary Figure 11c). Functional enrichment analysis showed enrichment of RING7 members in the ERAD pathway, protein deubiquitination in ubiquitin-dependent protein catabolism, and regulation of cell growth. Centrosomes and ubiquitin ligase complexes are enriched at the cellular component level with RING7 E3s. They mediate actin binding, unmethylated CpG binding, and transcription co-regulator activity (Fig. 4, Supplementary Figure 18). The PDB contains 142 structures covering 17 out of 27 RING7 proteins resolved in the PDB. We observed that seven proteins function as complexes, among which are FXL19, KDM2A, and KDM2B, all grouped in the KDM subfamily and probably serve as a substrate-recognition component of the SCF (SKP1-CUL1-F-box protein)-type E3 ubiquitin ligase complex. Most of the other RING7 family proteins are likely to function as standalone ligases, and the mode of action remains undetermined for six proteins (Supplementary Table 5). Analysis of the ESI network for this family revealed 109 unique E3-specific substrates and nine family-specific substrates (Supplementary Table 6). We identified $\sim$ 5000 small molecule binders for HDAC6, within the same family, small molecules targeting KDM proteins, and RNF31 could also be observed (Fig. 6a).

Note 10: RING8 family (TRIM+SPRY, TRIM−SPRY)

The RING8 family comprises 18 proteins predominantly classified as TRIM E3 ligases (Supplementary Figure 12a). These proteins contain either RING-type or B-box-type Zn-fingers as their catalytic domains (Supplementary Figure 12b). Within this family, two distinct subfamilies have been identified based on their domain architecture and sequence similarity. The first subfamily (TRIM+SPRY) encompasses approximately half of the proteins and is characterized by three specific domains: the SPRY domain, the COS domain, and the Fibronectin type III domain (IPR003961). The Fibronectin type III domain is a structural module featuring Arg-Gly-Asp (–RGD–) repeats, providing functional and structural versatility. The B30.2/SPRY

domain facilitates protein-protein interactions in these E3s, while the COS domain mediates microtubule association. The second subfamily (TRIM−SPRY) lacks these characteristic domains and demonstrates greater structural consistency among its members. This classification aligns with previously established domain-based categorizations of TRIM E3s. Additionally, RNF207, a RING-type RNF ligase with a Zn-finger B-box type and RING-type domain, is co-clustered with this family. Multiple sequence alignment (MSA) of the RING8 family revealed the conservation of the RING domain, B-box type, and B-box C-terminal domain (Supplementary Figure 12c). Functional analysis indicated that this family is enriched in specific biological processes, including suppression of viral release by the host, innate immune response, and positive regulation of autophagy. Additionally, key molecular functions such as transcription coregulator activity, ubiquitin-protein ligase activity, and protein homodimerization activity are significantly enriched in this family (Fig. 4, Supplementary Figure 18). The PDB contains 78 resolved structures, covering 16 of 18 RING8 E3 ligases. Among these, eight proteins are likely to function as standalone ligases, nine as components of complex ligases, and one remains unclassified (Supplementary Table 5). Examination of the E3-substrate interaction (ESI) network for this family identified 86 unique E3-specific substrates and 20 family-specific substrates (Supplementary Table 6). Additionally, a few small-molecule binders for TIF1A and TRIM33 were detected, which could potentially be developed into E3-targeting handles (Fig. 6a).

Note 11: RING9 family (Deltex, LNX-like, LON peptidase, Other RING)

The RING9 family comprises 69 E3 ligases with sparse and diverse InterPro domain annotations. The Zn-finger RING-type domain is often the primary annotated feature within this family (Supplementary Figure 13b). Based on domain architecture and sequence similarity, the family is further categorized into four distinct subfamilies (Supplementary Figure 13a). The first subfamily consists of five Deltex family members, characterized by the presence of the Deltex C-terminal domain (IPR039396), which contains an ADP-ribose-binding pocket and plays a key role in mediating cell-to-cell communication. Additionally, these proteins possess a WWE domain that recognizes poly-ADP-ribosylated substrates and plays a crucial role in DNA repair. These domain-associated functionalities align with the observed enrichment in biological processes such as the Notch signaling pathway and the cellular components associated with sites of DNA damage. The remaining three subfamilies exhibit diverse domain architectures. The subfamily termed "LNX-like" includes ligases such as CBL, Ligand of Numb protein-X (LNX), SH3RF, and NFX. Within this group, CBL ligases are distinguished by an N-terminal Cbl adaptor protein with a PTB domain (IPR024159). Members of the LNX family are characterized by PDZ domain repeats (IPR001478), which are typically found in signaling proteins localized to highly specialized sub-membranous sites. SH3 domain-containing ligases are implicated in apoptosis, whereas NFX ligases contain NF-X1-type repeats, highlighting diverse functional roles within this subfamily. The "Other RING" subfamily includes ligases that contain GB1/RHD3-type guanine nucleotide (G) binding domains (IPR030386), found in RN112 ligases, as well as N-terminal FHA domains, observed in CHFR and RNF8 ligases. Additionally, this subfamily includes ligases such as LRSM1 and BFAR, which feature a sterile alpha motif (SAM) domain. The "LON peptidase-like" subfamily includes E3 ligases such as LON peptidase, BRE, and VPS. LON peptidase ligases are notable for containing two RING-type Zn-finger domains and an N-terminal Lon protease domain, which is involved in the degradation of short-lived regulatory and damaged proteins. Other ligases in this group contain only the Zn-finger RING domain. This heterogeneity in sequence and domain composition is reflected in the multiple sequence alignment (MSA) of the family, which exhibits numerous gaps (Supplementary Figure 13c). The RING9 family is enriched in biological processes such as protein K6-linked ubiquitination, response to ionizing radiation, and endosomal vesicle fusion. Additionally, RING9 ligases are predominantly localized to the PML body, the site of DNA damage, and the ER quality control compartments. These ligases are also associated with molecular functions such as histone ubiquitin ligase activity and SH3 domain binding (Fig. 4, Supplementary Figure 18). The PDB contains 144 structures covering 32 out of 69 RING9 proteins. Most RING9 E3 ligases are likely to function as standalone ligases, with only five operating as part of a complex, while the remaining E3s remain unclassified (Supplementary Table 5). Analysis of the ESI network for this family revealed 296 unique E3-specific substrates and 66 family-specific substrates (Supplementary Table 6). Small molecule binders were identified specific to CBLB, which form 3 neat segregated clusters, indicating that a similar scaffold interacting with presumably the same binding site can be optimized for handle design (Supplementary Figure 22).

Note 12: RING10 family (NSD-like, UBR-like)

The RING10 family comprises 31 E3 ligases, each exceeding 1000 residues in length (Supplementary Figure 14b). The catalytic domains within this family include RING-, PHD-, UB-R, TAZ-, and C2H2-type Zn-finger domains. Based on domain architecture and sequence similarity, this family is subdivided into two distinct subfamilies (Supplementary Figure 14a). The first subfamily, "NSD-like," includes NSD1–3 (nuclear receptor-binding SET domain proteins), which are characterized by multiple functional domains such as the PWWP domain, Zn-finger PHD-type domain, SET domain, and NSD CYS-HIS domain. A defining feature of this subfamily is the repetition of Zn-finger PHD-type domains, which are frequently implicated in chromatin interactions. Notably, the SET and PHD-type Zn-finger domains are strongly associated with histone modification and methyltransferase activity,

highlighting their potential regulatory role in epigenetic processes. The second subfamily, "UBR-like," includes CHD, UBR, and BAZ1 ligases, along with other singletons. CHD ligases contain chromodomains, helicase superfamily 1/2 ATP-binding domains, CHD subfamily II-specific features, and SANT-like domains, suggesting roles in chromatin remodeling and gene regulation. The UBR ligases are distinguished by the UBR-like C-terminal domain (IPR044046), forming a prominent subgroup within this family. Ligases such as BAZ1 exhibit a multidomain architecture, including WSTF/Acf1/Cbp146 domains, DDT domains, WHIM1 and WHIM2 domains, PHD-type Zn-finger domains, bromodomains, Tudor domains, and BRCT domains, indicating multifunctional roles in chromatin interactions, DNA repair, and transcriptional regulation. Several members of this subfamily contain the Zn-finger PHD-finger domain, which is commonly found in nuclear proteins that interact with chromatin. The enrichment of various domain types within this subfamily suggests a role in chromatin remodeling, as further supported by the RING10 family's association with nuclear chromosomes and histone acetyltransferase complex cellular components, as well as chromatin-binding and histone-binding molecular functions. However, the heterogeneity of these subfamilies is evident from the multiple sequence alignment (MSA), which exhibits numerous gaps (Supplementary Figure 14c). RING10 members are enriched in biological processes such as epigenetic regulation of gene expression, protein polyubiquitination, and protein acetylation. They are localized to the nuclear speck, histone acetyltransferase complexes, and the PML body, where they mediate histone-binding activity, transcription coregulator activity, and chromatin binding (Fig. 4, Supplementary Figure 18). The PDB contains 296 resolved structures covering 21 out of 31 RING10 proteins. Most RING10 family proteins are likely to function as standalone ligases, with only three operating as part of a complex, while the remaining seventeen ligases remain unclassified (Supplementary Table 4). Analysis of the ESI network for this family revealed 31 unique E3-specific substrates and four family-specific substrates (Supplementary Table 5). Two E3 handles exist for UBR1. Our analysis uncovered new E3s binders targeting NSD2, CHD4, M3K1, CBP, EP300, BAZ1A, KAT6A, NSD3, and BRCA1. Several E3 binders target CBP and EP300, which are co-clustered. Several of these E3 binders are unique and form distinct clusters (KAT6A targeting) and could be developed into lead compounds (Fig. 6a,f).

Note 13: HECT1 family (NEDD4-like, HERC, Other HECT)

The HECT1 family contains 20 E3s (Supplementary Figure 15a) characterized by a C-terminal catalytic HECT domain (IPR000569) and one or two N-terminal domains (Supplementary Figure 15b). The HECT domain spans approximately 350 amino acids at the C-terminus (Supplementary Figure 15c) and is structured into two lobes: the N-lobe and the C-lobe. The N-lobe is responsible for binding to the E2 enzyme-ubiquitin complex, which facilitates the transfer of ubiquitin from E2 to the C-lobe through a thioester intermediate involving a crucial catalytic cysteine. Once ubiquitin is appropriately positioned on the C-lobe, it is transferred to the target protein. Based on their N-terminal domains, the HECT family is categorized into three main subfamilies: NEDD4-like, HERC, and other HECT. The "NEDD4-like" subfamily comprises 9 HECT proteins (NED4L, NEDD4, ITCH, WWP1-2, SMUF1-2, HECW1-2), mainly co-clustered according to sequence and domain architecture similarity, which feature the C2 (IPR000008) and WW (IPR001202) domains. The C2 domain aids in signal transduction and membrane trafficking in a calcium-dependent manner. It can also bind to phospholipids in a calcium-dependent manner, enabling membrane recruitment of the E3s. The WW domain contains two tryptophan residues, is involved in protein/protein interactions by recognizing proline-rich motifs (–PY– or –PPxY–) on target proteins, and is often associated with signal transduction proteins. The other 11 E3s forming the HERC and other HECT subfamilies possess either a different N-terminal domain or none. The HERC subfamily contains four small HERC E3s with no N-terminal domain annotations, and they display high intra-subfamily similarity on every metric layer. The two larger HERC1 and HERC2 are co-clustered with the HECT2 family. The HERC proteins feature the (RLD) RCC1/BLIP II domain (IPR000408), known to act as a guanine-nucleotide dissociation stimulator, while HERC1 and HERC2 have additional domains. The other HECT subfamily contains HECT proteins with one or two different N-terminal domains. HECD3 contains the APC10/doc domain (IPR004939), which may facilitate ubiquitination reactions with other RING domains. UBE3A features an N-terminal Zn-binding domain, or AZUL domain (IPR032353), likely playing a role in substrate recognition. UBE3C and UBE3B feature an IQ motif. AREL1 possesses a Filamine/ABP280 repeat (IPR017868). The HACE1 includes Ankyrin repeats (IPR002110) common to protein-protein interaction motifs. HECD2 does not have any N-terminal domains. The enrichment analysis shows the association of the HECT1 family with biological processes such as regulation of dendrite morphogenesis, cell growth, and protein K-63-linked ubiquitination (Fig. 4, Supplementary Figure 18). The PDB contains 80 resolved structures that cover 13 of 20 HECT1 proteins. Almost all HECT1 E3s are likely to function as standalone ligases; only four were found to operate as complex ligases (Supplementary Table 5). Analysis of the ESI network for this family revealed 270 unique E3-specific substrates and 433 family-specific substrates (Supplementary Table 6). SMUF1, SMUF2, and NEDD4 have E3 binders, which could be developed into lead compounds for E3 handle design (Fig. 6a).

Note 14: HECT2 family (HECT, KMT2-like)

The HECT2 family comprises 13 proteins organized into two subfamilies (Supplementary Figure 16a). The seven HECT domain-containing proteins are clustered into one subfamily, referred to as the "HECT" subfamily, while five RING ligases are grouped in the "KMT2-like" subfamily. A shared feature of these multi-domain E3 ligases is their enormous size, exceeding 2000 residues. Within the HECT subfamily, further subdivisions are observed: HERC1 and HERC2 form one subset, whereas the other HECT proteins, HECTD4 and HUWE1, comprise another subgroup. Both HERC1 and HERC2 possess HERC subfamily-specific RCC1/RLD domains, and HERC2 additionally includes CYT5 (IPR001199), MIB (IPR010606), and Znf (IPR000433) domains. HECTD4 contains a SPRY domain (IPR001870), which likely coordinates protein-protein interactions. HUWE1 features ARM repeats and UBA (IPR041918) and WWE (IPR041918) domains. The second subgroup includes UBR5, TRIPC, HECTD1, and CUL9 (an outlier in the RBR class). These proteins are relatively smaller and display multiple domains (Supplementary Figure 16b). HECTD1 contains Ankyrin repeats (IPR036770), SUN domain (IPR002110), and MIB (IPR010606) domains, while TRIPC includes both ARM and WWE domains. UBR5 is characterized by the E3 ubiquitin ligase EDD domain (IPR024725), along with RCC1/RLD, UBR box Zn-finger (IPR047503), and Poly(A)-binding protein C-terminal (PABC) domain (IPR002004). CUL9 (the outlier) contains a TRIAD domain at the C-terminal, and other domains such as DOC/APC10 and the Cullin Nedd8-2 domain (IPR001373) can act as scaffold proteins for other E3s by forming part of a multi-subunit ubiquitin complex. The "KMT2-like" subfamily clusters together 5 RING E3 ligases, which display variable Zn-finger RING- and PHD-type domains. Like the HECT proteins, MYCB2 also possesses an RCC1/RLD domain, while other proteins contain PHR, Filamin/ABP280 repeats, DOC, and B-box type domains. KMT2C and KMT2D feature multiple PHD-type Zn-finger domains, an HMG box, and an FYRN motif at their C-terminal. UBR4 has an N-terminal UBR box domain (IPR045841), followed by Zn-finger and WD40 repeats, concluding with a C-terminal UBR domain. The protein RN213 contains an AAA5 domain (IPR003593) and Zn-finger RING-type and RZ-type (IPR046439) domains. This family's enormous size and variable domain architectures contribute to a poor MSA (Supplementary Figure 16c). Members of the HECT2 family are enriched in biological processes related to the epigenetic regulation of gene expression and branched polyubiquitination. They also mediate guanyl-nucleotide exchange and protein ubiquitination (Fig. 4, Supplementary Figure 18). The PDB contains 53 structures covering 10 out of the 13 HECT2 E3s. HECT2 family members are likely to function as standalone proteins, supported by their considerable sequence lengths and multi-domain compositions, which align with their functional mode as independent proteins (Supplementary Table 5). Analysis of the ESI network for this family revealed 18 unique E3-specific substrates and one family-specific substrate (Supplementary Table 6). Currently, no small molecules explicitly target the HECT2 family.

Note 15: RBR family

The RBR family is organized into a single subfamily composed of 12 RBR E3 ligases, typically exceeding 700 residues in length. Members of this family are characterized by the catalytic TRIAD supra-domain (IPR044066), which comprises the RING1, BRcat (IBR), and Rcat (RING2) domains (Supplementary Figure 17a,b). This conserved architecture of domains is typical among eukaryotic RBR E3 ligases. The TRIAD domain is observed at the C-terminal region of RN216, RN217, RNF14, PRKN, and HOIL1, which are multi-domain proteins that also contain Ariadne, Parkin, RING Ubox-like zinc-binding, and Zn-finger RanBP2-type domains. Interestingly, the RBR cluster excludes CUL9 (UniProt Q8IWT3) and RNF31 (UniProt Q96EP0), which are clustered within the HECT2 and RING7 families. HHARI (ARI1) and TRIAD1 (ARI2) contain an Ariadne domain (IPR045840) at the C-terminal. RNF14 has an RWD domain (IPR006575) at the N-terminal. PRKN and HOIL1 include ubiquitin-like domains at the N-terminal, followed by Zn-finger domains—specifically, a RING/Ubox-like zinc-binding domain in PRKN and a RanBP2-type domain (IPR001876) in HOIL1. The MSA confirms the conserved TRIAD supradomain as the characteristic feature of this family (Supplementary Figure 17c). These ligases utilize a two-step catalytic mechanism where RING1 recognizes the E2-Ub conjugate, transfers the ubiquitin to a conserved catalytic cysteine on RING2 to form a thioester intermediate, and subsequently transfers the ubiquitin to the lysine residue of the substrate. Functionally, RBR ligases are involved in oncogenesis and tumor-suppressive roles. Enrichment analysis reveals associations with biological processes such as protein K6-linked ubiquitination, protein polyubiquitination, and general protein ubiquitination. They localize to ubiquitin ligase complexes, and their molecular functions include ubiquitin-conjugating enzyme binding, ubiquitin-like protein binding, and transcription coregulator activity (Fig. 4, Supplementary Figure 18). The PDB contains 38 structures covering 6 out of 12 RBR proteins. The plausible mode of action for RBR E3 ligases indicates that most function as standalone ligases, while PRKN, ARI1, and ARI2 may act as complex E3 ligases (Supplementary Table 5). Analysis of the ESI network for this family revealed 101 unique E3-specific substrates and 65 family-specific substrates (Supplementary Table 6). We identified one small molecule binder for HOIL1, which could be developed into an E3 handle for HOIL1 and repurposed for PRKN and other co-clustered E3s (Fig. 6a).

Note 16: Non-degradative functions of the human E3 ligome

The ubiquitin code signals and dictates different cellular outcomes. The functional diversity as a consequence of substrate ubiquitination is defined by the type of ubiquitin linkage and the structure of Ub-chains (*20, 52*). While K48-linked chains (Trash-tags) mark substrates for canonical proteasomal degradation are generic (all E3 families), a wide array of non-degradative roles are family-specific and linked directly to distinct enriched chain linkages (GO BP terms). K63-linked chains (Scaffold-tag; RING1, RING4, RING5, RING8, RING9, and HECT1) mediate signaling pathways, intracellular trafficking, autophagy, and immune responses (*14, 15, 32, 36*) by bringing proteins together to form functional complexes. K6-linked chains (Repair tag; RING9 and RBR) mediate DNA damage response, RNA–protein cross-link repair, and mitophagy (*8, 34, 61, 62*). K11-linked chains (Cycle-tag; RING4) mediate cell cycle control and the ERAD pathway in addition to proteasomal degradation (*59*).

Other family-specific biological processes enriched in our analysis are presumably mediated by alternative Ub-links. K29-linked chains (Recycling tag; RING9 and RING4) mediate lysosomal degradation (autophagy), Wnt signaling, and regulation of the apoptotic process (*16*). K33- linked chains (Traffick tag; RNF2, CBLB, ITCH) regulate cell signaling, immune response, and protein trafficking (*23, 33, 63*). M1-linkage (Alarm tag; RNF31, HOIL1) predominantly regulates NF-κB signaling, cell death regulation, protein quality control, and immune signaling (*19, 55*). Additionally, branched linkages also mediate NF-κB signaling, ERAD, Cyclin B1 regulation, mitotic progression, and cell cycle response (*18, 21*). Emerging evidence shows that even K48-linked chains (RING4) regulate DNA damage and NF-kB signaling via branched ubiquitination (*37, 44*). Overall, we find that the ubiquitin code is preserved in our analysis and reflected in our classification of E3 families. This functional diversity positions the E3 ligome as a central hub for maintaining cellular homeostasis, expanding its role well beyond the classical proteasomal degradation paradigm.

Note 17: Guidelines for harnessing the human E3 ligome

Here, we outline practical guidelines for utilizing the proposed human E3 ligome classification for ubiquitin data interpretation, screening studies, and therapeutic design. Broadly, the following guidelines are derived from (i) mapping the E3 ligome structure onto high-throughput datasets and analyzing overlap patterns (coverage; Fig. 3), (ii) integration of E3 classification with external data to map the functional segregation pattern (Fig. 4), enzyme–substrate networks (Fig. 5), and small molecule interaction landscape (Fig. 6), and (iii) design of new experiments using the classification model as a hypothesis generator for testing and validation.

Informative annotations

In the analysis of high-throughput (HTP) screening data, the proposed E3 ligome structure will aid in annotating new candidates and identifying family- and subfamily-specific enrichment patterns. Since the E3 families and subfamilies obtained here are tightly associated with specific biological pathways (Fig. 4), family-level up- or down-regulation can aid in inferring pathway activation or repression under defined screening/experimental conditions. Further, it can also aid in mapping differentially expressed or modified substrates (new targets) to corresponding E3 ligases.

Analyzing proteomics screens

Mapping the current E3 ligome structure onto ubiquitination mass spectrometry (MS) data can lead to validation of candidate E3s and the detection of previously uncharacterized enzymes. MS-based HTP ubiquitination studies typically rely on the enrichment of diGly-remnant peptides (K–$\mathcal{E}$–GG). However, these signatures are not fully specific to ubiquitination, as they can also arise from other ubiquitin-like modifiers such as ISG15 and NEDD8. Moreover, diGly proteomics cannot capture linear ubiquitin linkages, limiting its ability to comprehensively map ubiquitination events. The ligome structure provides a valuable framework to integrate advanced substrate identification methods such as Ub–POD—a ubiquitin-specific, proximity- and orientation-dependent labeling technique (*41*). Combining both approaches will enable systematic and more precise mapping of ESIs, revealing family- or subfamily-specific ubiquitination networks and functional dependencies. Further, discrepancies between substrate ubiquitination patterns, E3 abundance, and curated E3-substrate interaction maps obtained in this study can be used to prioritize candidate E3s for functional validation. When a well-known E3 is down-regulated or knocked out but the substrate remains ubiquitinated, closely related upregulated E3s (inferred from the E3 ligome) could represent a candidate set for follow-up testing to assess compensatory mechanisms. For instance, using the lung squamous-cell carcinoma molecular subgroups identified by multi-omics clustering (*49*), active pathway differences across subgroups can be identified by mapping redundant or unique E3s.

Identification of E3–degron pairs

The E3 ligome framework can aid in integrating and interpreting degron mapping efforts. For example, a recent study (*64*) identified 16 distinct E3–degron pairs using global protein stability (GPS) profiling and mutagenesis. As the repertoire of E3-degron pairs expands, our classification can contextualize whether degrons are shared (degenerate or redundant) across family/sub-family members or E3-specific (functionally specialized), helping define functional hierarchies within the ubiquitination system. Further, newly identified degrons can be used to screen the unmapped fraction of the ubiquitinated proteome and expand our current ESI network.

Designing family- and subfamily-level E3 screens

The current classification allows rational design of mechanism-based HTP assays at the family or subfamily level. Approaches such as activated ubiquitin surrogates, proximity labeling, or E2 Ub conjugate trapping can be implemented in parallel across the proposed E3 family members (*17,41,46*). Further, quantitative comparisons with inactive mutants or external controls (other E3s, outside the family of interest) will allow direct estimation of background activity and family-specific enrichment. Parallel assays using catalytic mutants, activity-based probes, or substrate-trapping variants can identify substrates determined by shared catalytic mechanisms rather than by binding alone (*5,25,58*). Comparative enrichment analyses can reveal mechanistic divergence among E3s. For instance, RING2 family (MARCH) ligases may preferentially modify exposed lysines on the cytosolic loops of membrane proteins; membrane fractionation and crosslinking experiments can therefore be used to identify family-specific substrates and adaptors (*2*).

Analyzing CRISPR-based genetic screens

E3 subfamily annotations can serve as analytical features to interpret dependency profiles across cell lines and experimental conditions (*54*). Clustering genes by genetic interaction (GI) correlations, such as co-dependency or co-sensitization, can reveal functional modules within subfamilies and identify non-redundant E3s. As proof-of-principle for family-level analysis of genetic screens, we demonstrated concordance between CRISPR–Cas9 dropout data and DepMap dependencies (Fig. 4a–c and Supplementary Figure 18a).

Identification of context-specific roles of E3s

Combining the ligome classification with single-cell or spatial transcriptomics can identify context-specific E3s, e.g., distinguishing tumor-compartment ligases from those active in the immune microenvironment. CRISPR-based studies (*54*) defining E3–substrate relationships can be extended using this framework to design targeted screens testing complex versus standalone E3 modalities (Fig. 5), and define the roles of adaptors, receptors, or scaffolds in substrate specificity.

Phenotypic profiling of E3s

E3 family annotations can enhance analysis of phenotypic data, such as cell-painting or live-cell imaging assays (*56*). By correlating E3 perturbations with morphological features or trajectory signatures of cells, it may be possible to infer pathway membership and identify candidate E3–substrate pairs associated with defined phenotypes.

Small-molecule screens and PROTAC design

Mapping E3 subfamilies with known small-molecule binders, UMAP clusters (inferred similarity on chemical landscape), or pocket similarities (e.g., ELIOT overlap (Figs. 6e–g) can enable identification of tractable ligases and rational repurposing of E3 handles across related E3 ligase families or nearest neighbors. HTP screens for one E3 can identify chemical matter that also engages structurally related E3s within the same sub-family, due to shared structural and pocket features. When active compounds are identified in small-molecule or phenotypic screens, integrating E3–substrate/adaptor–scaffold relationships can help trace causal mechanisms and link chemical space with functional E3 modules. Often, targeting the natural *in vivo* substrates (POI) of cognate E3s via PROTACs works as the best strategy with the least interference. Altogether, this will enable the design of family-informed degradation strategies.

Supplementary References

[1] Yoshino Akizuki, Stephanie Kaypee, Fumiaki Ohtake, and Fumiyo Ikeda. The emerging roles of non-canonical ubiquitination in proteostasis and beyond. *Journal of Cell Biology*, 223(5), March 2024.

[2] Orhi Barroso-Gomila, Laura Merino-Cacho, Veronica Muratore, Coralia Perez, Vincenzo Taibi, Elena Maspero, Mikel Azkargorta, Ibon Iloro, Fredrik Trulsson, Alfred C. O. Vertegaal, Ugo Mayor, Felix Elortza, Simona Polo, Rosa Barrio, and James D. Sutherland. Bioe3 identifies specific substrates of ubiquitin e3 ligases. *Nature Communications*, 14(1), November 2023.

[3] Sabrina Berkamp, Siavash Mostafavi, and Carsten Sachse. Structure and function of p62/sqstm1 in the emerging framework of phase separation. *The FEBS Journal*, 288(24):6927–6941, December 2020.

[4] Lori Buetow and Danny T. Huang. Structural insights into the catalysis and regulation of e3 ubiquitin ligases. *Nature Reviews Molecular Cell Biology*, 17(10):626–642, August 2016.

[5] Robert Byrne, Thomas Mund, and Julien D. F. Licchesi. Activity-based probes for hect e3 ubiquitin ligases. *ChemBioChem*, 18(14):1415–1427, June 2017.

[6] Umar-Faruq Cajee, Rodney Hull, and Monde Ntwasa. Modification by ubiquitin-like proteins: Significance in apoptosis and autophagy pathways. *International Journal of Molecular Sciences*, 13(9):11804–11831, September 2012.

[7] The UniProt Consortium. UniProt: the Universal Protein Knowledgebase in 2023. *Nucleic Acids Research*, 51(D1):D523–D531, 11 2022.

[8] Christian N. Cunningham, Joshua M. Baughman, Lilian Phu, Joy S. Tea, Christine Yu, Mary Coons, Donald S. Kirkpatrick, Baris Bingol, and Jacob E. Corn. Usp30 and parkin homeostatically regulate atypical ubiquitin chains on mitochondria. *Nature Cell Biology*, 17(2):160–169, January 2015.

[9] Raymond J. Deshaies and Claudio A.P. Joazeiro. Ring domain e3 ubiquitin ligases. *Annual Review of Biochemistry*, 78(1):399–434, 2009.

[10] Ivan Dikic and Brenda A. Schulman. An expanded lexicon for the ubiquitin code. *Nature Reviews Molecular Cell Biology*, 24(4):273–287, October 2022.

[11] Gunnar Dittmar and Konstanze F. Winklhofer. Linear ubiquitin chains: Cellular functions and strategies for detection and quantification. *Frontiers in Chemistry*, 7, January 2020.

[12] Kasidy K. Dobish, Karli J. Wittorf, Samantha A. Swenson, Dalton C. Bean, Catherine M. Gavile, Nicholas T. Woods, Gargi Ghosal, R. Katherine Hyde, and Shannon M. Buckley. Fbxo21 mediated degradation of p85α regulates proliferation and survival of acute myeloid leukemia. *Leukemia*, 37(11):2197–2208, September 2023.

[13] Jane Dudley-Fraser, Diego Esposito, Katherine A. McPhie, Coltrane Morley-Williams, Tania Auchynnikava, and Katrin Rittinger. Identification of ring e3 pseudoligases in the trim protein family. *Nature Communications*, 16(1), April 2025.

[14] Anna Dósa and Tamás Csizmadia. The role of k63-linked polyubiquitin in several types of autophagy. *Biologia Futura*, 73(2):137–148, May 2022.

[15] Zoi Erpapazoglou, Olivier Walker, and Rosine Haguenauer-Tsapis. Versatile roles of k63-linked ubiquitin chains in trafficking. *Cells*, 3(4):1027–1088, November 2014.

[16] Cong Fei, Zhenfei Li, Chen Li, Yuelei Chen, Zhangcheng Chen, Xiaoli He, Li Mao, Xin Wang, Rong Zeng, and Lin Li. Smurf1-mediated lys29-linked nonproteolytic polyubiquitination of axin negatively regulates wnt/β-catenin signaling. *Molecular and Cellular Biology*, 33(20):4095–4105, October 2013.

[17] Tyler G. Franklin and Jonathan N. Pruneda. A high-throughput assay for monitoring ubiquitination in real time. *Frontiers in Chemistry*, 7, December 2019.

[18] Michael E. French, Chad F. Koehler, and Tony Hunter. Emerging functions of branched ubiquitin chains. *Cell Discovery*, 7(1), January 2021.

[19] Björn Gerlach, Stefanie M. Cordier, Anna C. Schmukle, Christoph H. Emmerich, Eva Rieser, Tobias L. Haas, Andrew I. Webb, James A. Rickard, Holly Anderton, Wendy W.-L. Wong, Ueli Nachbur, Lahiru Gangoda, Uwe Warnken, Anthony W. Purcell, John Silke, and Henning Walczak. Linear ubiquitination prevents inflammation and regulates immune signalling. *Nature*, 471(7340):591–596, March 2011.

[20] Maria Gonzalez-Santamarta, Corentin Bouvier, Manuel S. Rodriguez, and Wendy Xolalpa. Ubiquitin-chains dynamics and its role regulating crucial cellular processes. *Seminars in Cell & Developmental Biology*, 132:155–170, December 2022.

[21] Diane L. Haakonsen and Michael Rape. Branching out: Improved signaling by heterotypic ubiquitin chains. *Trends in Cell Biology*, 29(9):704–716, September 2019.

[22] Youngwoong Han, Hodong Lee, Jong C Park, and Gwan-Su Yi. E3net: a system for exploring e3-mediated regulatory networks of cellular functions. *Molecular & cellular proteomics*, 11(4), 2012.

[23] Haining Huang, Myung-shin Jeon, Lujian Liao, Chun Yang, Chris Elly, John R. Yates, and Yun-Cai Liu. K33-linked polyubiquitination of t cell receptor-ζ regulates proteolysis-independent t cell signaling. *Immunity*, 33(1):60–70, July 2010.

[24] Xuejun Jin, Hong Ri Jin, Haeng Sun Jung, Se Jeong Lee, Jeong-Hyung Lee, and Jung Joon Lee. An atypical e3 ligase zinc finger protein 91 stabilizes and activates nf-κb-inducing kinase via lys63-linked ubiquitination. *Journal of Biological Chemistry*, 285(40):30539–30547, October 2010.

[25] Victoria N. Jordan, Alban Ordureau, and Heeseon An. Identifying e3 ligase substrates with quantitative degradation proteomics. *ChemBioChem*, 24(16), July 2023.

[26] Yogesh Kulathu, Francisco J. Garcia, Tycho E. T. Mevissen, Martin Busch, Nadia Arnaudo, Kate S. Carroll, David Barford, and David Komander. Regulation of a20 and other otu deubiquitinases by reversible oxidation. *Nature Communications*, 4(1), March 2013.

[27] Laurent Le Cam, Laëtitia K. Linares, Conception Paul, Eric Julien, Matthieu Lacroix, Elodie Hatchi, Robinson Triboulet, Guillaume Bossis, Ayelet Shmueli, Manuel S. Rodriguez, Olivier Coux, and Claude Sardet. E4f1 is an atypical ubiquitin ligase that modulates p53 effector functions independently of degradation. *Cell*, 127(4):775–788, November 2006.

[28] Jennifer Lee and Pengbo Zhou. Dcafs, the missing link of the cul4-ddb1 ubiquitin ligase. *Molecular cell*, 26(6):775–780, 2007.

[29] Wei Li, Mario H. Bengtson, Axel Ulbrich, Akio Matsuda, Venkateshwar A. Reddy, Anthony Orth, Sumit K. Chanda, Serge Batalov, and Claudio A. P. Joazeiro. Genome-wide and functional annotation of human e3 ubiquitin ligases identifies mulan, a mitochondrial e3 that regulates the organelle's dynamics and signaling. *PLOS ONE*, 3(1):1–14, 01 2008.

[30] Zhongyan Li, Siyu Chen, Jhih-Hua Jhong, Yuxuan Pang, Kai-Yao Huang, Shangfu Li, and Tzong-Yi Lee. UbiNet 2.0: a verified, classified, annotated and updated database of E3 ubiquitin ligase–substrate interactions. *Database*, 2021, January 2021.

[31] Lihua Liu, David R Damerell, Leonidas Koukouflis, Yufeng Tong, Brian D Marsden, and Matthieu Schapira. UbiHub: a data hub for the explorers of ubiquitination pathways. *Bioinformatics*, 35(16):2882–2884, 01 2019.

[32] Pengda Liu, Wenjian Gan, Siyuan Su, Arthur V. Hauenstein, Tian-min Fu, Bradley Brasher, Carsten Schwerdtfeger, Anthony C. Liang, Ming Xu, and Wenyi Wei. K63-linked polyubiquitin chains bind to dna to facilitate dna damage repair. *Science Signaling*, 11(533), June 2018.

[33] Shuo Liu, Minghong Jiang, Wendie Wang, Wei Liu, Xiaoqi Song, Zhongfei Ma, Shikun Zhang, Lun Liu, Yin Liu, and Xuetao Cao. Nuclear rnf2 inhibits interferon function by promoting k33-linked stat1 disassociation from dna. *Nature Immunology*, 19(1):41–52, November 2017.

[34] Chi-Sheng Lu, Lan N. Truong, Aaron Aslanian, Linda Z. Shi, Yongjiang Li, Patty Yi-Hwa Hwang, Kwi Hye Koh, Tony Hunter, John R. Yates, Michael W. Berns, and Xiaohua Wu. The ring finger protein rnf8 ubiquitinates nbs1 to promote dna double-strand break repair by homologous recombination. *Journal of Biological Chemistry*, 287(52):43984–43994, December 2012.

[35] Peter D Mabbitt, Andrea Loreto, Marc-André Déry, Adam J Fletcher, Mathew Stanley, Kuan-Chuan Pao, Nicola T Wood, Michael P Coleman, and Satpal Virdee. Structural basis for RING-Cys-Relay E3 ligase activity and its role in axon integrity. *Nat. Chem. Biol.*, 16(11):1227–1236, November 2020.

[36] Charitha Madiraju, Jeffrey P. Novack, John C. Reed, and Shu-ichi Matsuzawa. K63 ubiquitination in immune signaling. *Trends in Immunology*, 43(2):148–162, February 2022.

[37] Frédérick A Mallette and Stéphane Richard. K48-linked ubiquitination and protein degradation regulate 53bp1 recruitment at dna damage sites. *Cell Research*, 22(8):1221–1223, April 2012.

[38] Anna Mattioni, Luisa Castagnoli, and Elena Santonico. Rnf11 at the crossroads of protein ubiquitination. *Biomolecules*, 10(11):1538, November 2020.

[39] Barbara Medvar, Viswanathan Raghuram, Trairak Pisitkun, Abhijit Sarkar, and Mark A Knepper. Comprehensive database of human e3 ubiquitin ligases: application to aquaporin-2 regulation. *Physiological genomics*, 48(7):502–512, 2016.

[40] Yiwei Mi, Lu Yan, Yu Wu, and Yufang Zheng. Deficiency of ube3d in mice leads to severe embryonic abnormalities and disrupts the mrna of homeobox genes via cpsf3. *Cell Death Discovery*, 11(1), March 2025.

[41] Urbi Mukhopadhyay, Sophie Levantovsky, Teresa Maria Carusone, Sarah Gharbi, Frank Stein, Christian Behrends, and Sagar Bhogaraju. A ubiquitin-specific, proximity-based labeling approach for the identification of ubiquitin ligase substrates. *Science Advances*, 10(32), August 2024.

[42] Koji Nomura, Marta Klejnot, Dominika Kowalczyk, Andreas K Hock, Gary J Sibbet, Karen H Vousden, and Danny T Huang. Structural analysis of mdm2 ring separates degradation from regulation of p53 transcription activity. *Nature Structural & Molecular Biology*, 24(7):578–587, May 2017.

[43] Melanie D. Ohi, Craig W. Vander Kooi, Joshua A. Rosenberg, Walter J. Chazin, and Kathleen L. Gould. Structural insights into the u-box, a domain associated with multi-ubiquitination. *Nature Structural & Molecular Biology*, 10(4):250–255, March 2003.

[44] Fumiaki Ohtake, Yasushi Saeki, Satoshi Ishido, Jun Kanno, and Keiji Tanaka. The k48-k63 branched ubiquitin chain regulates nf-κb signaling. *Molecular Cell*, 64(2):251–266, October 2016.

[45] Rose Oughtred, Jennifer Rust, Christie Chang, Bobby-Joe Breitkreutz, Chris Stark, Andrew Willems, Lorrie Boucher, Genie Leung, Nadine Kolas, Frederick Zhang, Sonam Dolma, Jasmin Coulombe-Huntington, Andrew Chatr-aryamontri, Kara Dolinski, and Mike Tyers. The biogrid database: A comprehensive biomedical resource of curated protein, genetic, and chemical interactions. *Protein Science*, 30(1):187–200, 2021.

[46] Hazel F O'Connor, Nancy Lyon, Justin W Leung, Poonam Agarwal, Caleb D Swaim, Kyle M Miller, and Jon M Huibregtse. Ubiquitin-activated interaction traps (¡scp¿ubait¡/scp¿ s) identify e3 ligase binding partners. *EMBO reports*, 16(12):1699–1712, October 2015.

[47] Hong Peng, Jiao Yang, Guangyi Li, Qing You, Wen Han, Tianrang Li, Daming Gao, Xiaoduo Xie, Byung-Hoon Lee, Juan Du, Jian Hou, Tao Zhang, Hai Rao, Ying Huang, Qinrun Li, Rong Zeng, Lijian Hui, Hongyan Wang, Qin Xia, Xuemin Zhang, Yongning He, Masaaki Komatsu, Ivan Dikic, Daniel Finley, and Ronggui Hu. Ubiquitylation of p62/sequestosome1 activates its autophagy receptor function and controls selective autophagy upon ubiquitin stress. *Cell Research*, 27(5):657–674, March 2017.

[48] Nicholas Purser, Ishita Tripathi-Giesgen, Jerry Li, Daniel C. Scott, Daniel Horn-Ghetko, Kheewoong Baek, Brenda A. Schulman, Arno F. Alpi, and Gary Kleiger. Catalysis of non-canonical protein ubiquitylation by the arih1 ubiquitin ligase. *Biochemical Journal*, 480(22):1817–1831, November 2023.

[49] Shankha Satpathy, Karsten Krug, Pierre M. Jean Beltran, Sara R. Savage, Francesca Petralia, Chandan Kumar-Sinha, Yongchao Dou, Boris Reva, M. Harry Kane, Shayan C. Avanessian, Suhas V. Vasaikar, Azra Krek, Jonathan T. Lei, Eric J. Jaehnig, Tatiana Omelchenko, Yifat Geffen, Erik J. Bergstrom, Vasileios Stathias, Karen E. Christianson, David I. Heiman, Marcin P. Cieslik, Song Cao, Xiaoyu Song, Jiayi Ji, Wenke Liu, Kai Li, Bo Wen, Yize Li, Zeynep H. Gümüş, Myvizhi Esai Selvan, Rama Soundararajan, Tanvi H. Visal, Maria G. Raso, Edwin Roger Parra, Özgün Babur, Pankaj Vats, Shankara Anand, Tobias Schraink, MacIntosh Cornwell, Fernanda Martins Rodrigues, Houxiang Zhu, Chia-Kuei Mo, Yuping Zhang, Felipe da Veiga Leprevost, Chen Huang, Arul M. Chinnaiyan, Matthew A. Wyczalkowski, Gilbert S. Omenn, Chelsea J. Newton, Stephan Schurer, Kelly V. Ruggles, David Fenyö, Scott D. Jewell, Mathangi Thiagarajan, Mehdi Mesri, Henry Rodriguez, Sendurai A. Mani, Namrata D. Udeshi, Gad Getz, James Suh, Qing Kay Li, Galen Hostetter, Paul K. Paik, Saravana M. Dhanasekaran, Ramaswamy Govindan, Li Ding, Ana I. Robles, Karl R. Clauser, Alexey I. Nesvizhskii, Pei Wang, Steven A. Carr, Bing Zhang, D.R. Mani, Michael A. Gillette, Alex Green, Alfredo Molinolo, Alicia Francis, Amanda G. Paulovich, Andrii Karnuta, Antonio Colaprico, Barbara Hindenach, Barbara L. Pruetz, Bartosz Kubisa, Brian J. Druker, Carissa Huynh, Charles A. Goldthwaite, Chet Birger, Christopher R. Kinsinger, Corbin D. Jones, Dan Rohrer, Dana R. Valley, Daniel W. Chan, David Chesla, Donna Hansel, Elena V. Ponomareva, Elizabeth Duffy, Eric Burks, Eric E. Schadt, Eugene S. Fedorov, Eunkyung An, Fei Ding, George D. Wilson, Harsh Batra, Hui Zhang, Jennifer E. Maas, Jennifer Eschbacher, Karen A. Ketchum, Karin D. Rodland, Katherine A. Hoadley, Kei Suzuki, Ki Sung Um, Liqun Qi, Lori Bernard, Maciej Wiznerowicz, Ma lgorzata Wojtyś, Marcin J. Domagalski, Matthew J. Ellis, Maureen A. Dyer, Melissa Borucki, Meenakshi Anurag, Michael J. Birrer, Midie Xu, Mikhail Krotevich, Nancy Roche, Nathan J. Edwards, Negin Vatanian, Neil R. Mucci, Nicollette Maunganidze, Nikolay Gabrovski, Olga Potapova, Oluwole Fadare, Pamela Grady, Peter B. McGarvey, Pushpa Hariharan, Ratna R. Thangudu, Rebecca Montgomery, Renganayaki Pandurengan, Richard D. Smith, Robert J. Welsh, Sailaja Mareedu, Samuel H. Payne, Sandra Cottingham, Shilpi Singh, Shirley X. Tsang, Shuang Cai, Stacey Gabriel, Tao Liu, Tara Hiltke, Tanmayi Vashist, Thomas Bauer, Volodymyr Sovenko, Warren G. Tourtellotte, Weiping Ma, William Bocik, Wohaib Hasan, Xiaojun Jing, Ximing Tang, Yuxing Liao, Yvonne, Shutack, Zhen Zhang, and Ziad Hanhan. A proteogenomic portrait of lung squamous cell carcinoma. *Cell*, 184(16):4348–4371.e40, August 2021.

[50] Daniel C. Scott, Sagar Chittori, Nicholas Purser, Moeko T. King, Samuel A. Maiwald, Kelly Churion, Amanda Nourse, Chan Lee, Joao A. Paulo, Darcie J. Miller, Stephen J. Elledge, J. Wade Harper, Gary Kleiger, and Brenda A. Schulman. Structural basis for c-degron selectivity across klhdcx family e3 ubiquitin ligases. *Nature Communications*, 15(1), November 2024.

[51] Gregory Segala, Marcela A. Bennesch, Nastaran Mohammadi Ghahhari, Deo Prakash Pandey, Pablo C. Echeverria, François Karch, Robert K. Maeda, and Didier Picard. Vps11 and vps18 of vps-c membrane traffic complexes are e3 ubiquitin ligases and fine-tune signalling. *Nature Communications*, 10(1), April 2019.

[52] Xiangpeng Sheng, Zhixiong Xia, Hanting Yang, and Ronggui Hu. The ubiquitin codes in cellular stress responses. *Protein & Cell*, 15(3):157–190, July 2023.

[53] Rakesh Kumar Singh, Melanie Gonzalez, Marie-Helene Miquel Kabbaj, and Akash Gunjan. Novel e3 ubiquitin ligases that regulate histone protein levels in the budding yeast saccharomyces cerevisiae. *PLoS ONE*, 7(5):e36295, May 2012.

[54] Richard T. Timms, Elijah L. Mena, Yumei Leng, Mamie Z. Li, Iva A. Tchasovnikarova, Itay Koren, and Stephen J. Elledge. Defining e3 ligase–substrate relationships through multiplex crispr screening. *Nature Cell Biology*, 25(10):1535–1545, September 2023.

[55] F. Tokunaga. Linear ubiquitination-mediated nf- b regulation and its related disorders. *Journal of Biochemistry*, 154(4):313–323, August 2013.

[56] Maria-Anna Trapotsi, Elizabeth Mouchet, Guy Williams, Tiziana Monteverde, Karolina Juhani, Riku Turkki, Filip Miljković, Anton Martinsson, Lewis Mervin, Kenneth R. Pryde, Erik Müllers, Ian Barrett, Ola Engkvist, Andreas Bender, and Kevin Moreau. Cell morphological profiling enables high-throughput screening for proteolysis targeting chimera (protac) phenotypic signature. *ACS Chemical Biology*, 17(7):1733–1744, July 2022.

[57] Xiangyi S. Wang, Thomas R. Cotton, Sarah J. Trevelyan, Lachlan W. Richardson, Wei Ting Lee, John Silke, and Bernhard C. Lechtenberg. The unifying catalytic mechanism of the ring-between-ring e3 ubiquitin ligase family. *Nature Communications*, 14(1), January 2023.

[58] Masashi Watanabe, Yasushi Saeki, Hidehisa Takahashi, Fumiaki Ohtake, Yukiko Yoshida, Yusuke Kasuga, Takeshi Kondo, Hiroaki Yaguchi, Masanobu Suzuki, Hiroki Ishida, Keiji Tanaka, and Shigetsugu Hatakeyama. A substrate-trapping strategy to find e3 ubiquitin ligase substrates identifies parkin and trim28 targets. *Communications Biology*, 3(1), October 2020.

[59] Katherine E. Wickliffe, Adam Williamson, Hermann-Josef Meyer, Aileen Kelly, and Michael Rape. K11-linked ubiquitin chains as novel regulators of cell division. *Trends in Cell Biology*, 21(11):656–663, November 2011.

[60] Karli J. Wittorf, Kasidy K. Weber, Samantha A. Swenson, and Shannon M. Buckley. Ubiquitin e3 ligase fbxo21 regulates cytokine-mediated signaling pathways, but is dispensable for steady-state hematopoiesis. *Experimental Hematology*, 114:33–42.e3, October 2022.

[61] Foon Wu-Baer, Karen Lagrazon, Wei Yuan, and Richard Baer. The BRCA1/BARD1 heterodimer assembles polyubiquitin chains through an unconventional linkage involving lysine residue K6 of ubiquitin. *J. Biol. Chem.*, 278(37):34743–34746, September 2003.

[62] Bo Yang, Jinyong Pei, Chen Lu, Yi Wang, Mengyang Shen, Xiao Qin, Yulu Huang, Xi Yang, Xin Zhao, Shujun Ma, Zhishan Song, Yinming Liang, Hui Wang, and Jie Wang. Rnf144a promotes antiviral responses by modulating sting ubiquitination. *EMBO reports*, 24(12), November 2023.

[63] Wei-Chien Yuan, Yu-Ru Lee, Shu-Yu Lin, Li-Ying Chang, Yen Pei Tan, Chin-Chun Hung, Jean-Cheng Kuo, Cheng-Hsin Liu, Mei-Yao Lin, Ming Xu, Zhijian J. Chen, and Ruey-Hwa Chen. K33-linked polyubiquitination of coronin 7 by cul3-klhl20 ubiquitin e3 ligase regulates protein trafficking. *Molecular Cell*, 54(4):586–600, May 2014.

[64] Zhiqian Zhang, Brandon Sie, Aiquan Chang, Yumei Leng, Christopher Nardone, Richard T. Timms, and Stephen J. Elledge. Elucidation of e3 ubiquitin ligase specificity through proteome-wide internal degron mapping. *Molecular Cell*, 83(18):3377–3392.e6, September 2023.
